# Novel Au Carbene Complexes as Promising Multi-Target Agents in Breast Cancer Treatment

**DOI:** 10.3390/ph15050507

**Published:** 2022-04-21

**Authors:** Jessica Ceramella, Annaluisa Mariconda, Marco Sirignano, Domenico Iacopetta, Camillo Rosano, Alessia Catalano, Carmela Saturnino, Maria Stefania Sinicropi, Pasquale Longo

**Affiliations:** 1 Department of Pharmacy, Health and Nutritional Sciences, University of Calabria, Via P. Bucci, 87036 Arcavacata di Rende, Italy; jessica.ceramella@unical.it (J.C.); s.sinicropi@unical.it (M.S.S.); 2 Department of Science, University of Basilicata, Viale dell’Ateneo Lucano 10, 85100 Potenza, Italy; annaluisa.mariconda@unibas.it (A.M.); carmela.saturnino@unibas.it (C.S.); 3 Department of Chemistry and Biology, University of Salerno, Via Giovanni Paolo II, 132, 84084 Fisciano, Italy; msirignano@unisa.it (M.S.); plongo@unisa.it (P.L.); 4 U.O. Proteomica e Spettrometria di Massa, IRCCS Ospedale Policlinico San Martino, Largo R. Benzi 10, 1632 Genova, Italy; 5 Department of Pharmacy-Drug Sciences, University of Bari “Aldo Moro”, 70126 Bari, Italy; alessia.catalano@uniba.it

**Keywords:** *N*-heterocyclic carbenes, human topoisomerases, actin, docking studies, breast cancer cells, multi-target agents

## Abstract

Over the past decade, metal complexes based on *N*-heterocyclic carbenes (NHCs) have attracted great attention due to their wide and exciting applications in material sciences and medicinal chemistry. In particular, the gold-based complexes are the focus of research efforts for the development of new anticancer compounds. Literature data and recent results, obtained by our research group, reported the design, the synthesis and the good anticancer activity of some silver and gold complexes with NHC ligands. In particular, some of these complexes were active towards some breast cancer cell lines. Considering this evidence, here we report some new Au-NHC complexes prepared in order to improve solubility and biological activity. Among them, the compounds **1** and **6** showed an interesting anticancer activity towards the breast cancer MDA-MB-231 and MCF-7 cell lines, respectively. In addition, in vitro and in silico studies demonstrated that they were able to inhibit the activity of the human topoisomerases I and II and the actin polymerization reaction. Moreover, a downregulation of vimentin expression and a reduced translocation of NF-kB into the nucleus was observed. The interference with these vital cell structures induced breast cancer cells’ death by triggering the extrinsic apoptotic pathway.

## 1. Introduction

Over the past decade, *N*-heterocyclic carbenes (NHCs) attracted the attention of several researchers because of their versatility and numerous applications. Their ability to bind various classes of substituents, including transition metals, made them suitable ligands for fast and efficient drug design [1]. Traditionally, the NHC–metal complexes play a very important role in material sciences as conducting polymeric, luminescent and liquid crystalline materials and also as efficient catalysts [2].

More recently, they have been considered with success for other applications including medicinal chemistry. In particular, the silver *N*-heterocyclic carbenes were employed as antimicrobial agents due to the ability of the silver cation to interact with the membrane or with the thiol groups of bacterial enzymes [3,4]. Instead, the gold complexes were employed in arthritis and cancer treatment, exerting multiple mechanisms of action. These latter represent the focus of research efforts for the development of new anticancer compounds, due to their ability to induce mitochondrial or DNA damage, to act as enzymes’ inhibitors, such as for the thioredoxin reductase, and to interfere with kinases, phosphatases, topoisomerases and the microtubules dynamic [5,6,7,8,9].

A considerable amount of literature data reported the good anticancer activity of some metal *N*-heterocyclic complexes and many gold-NHC complex derivatives, and have been published for their interesting antitumor activity in the micromolar or submicromolar range, due to their antimitochondrial properties, as well as their ability to inhibit several enzymes, to induce cell death by apoptosis and cause an increase in reactive oxygen species [10,11,12]. Additionally, promising results have been recently obtained from some of us concerning the anticancer properties of several silver and gold NHC complexes [5,7,13]. The antitumor activity of these metal complexes was evaluated towards different cancer cell lines and some of them, mostly gold complexes, possessed a good anticancer activity against MCF-7 and MDA-MB-231 breast cancer cell lines, with IC_50_ values in the micromolar range.

These scientific evidences might be seminal in the fight against breast cancer, which represents the most frequently diagnosed type of cancer among women of all ages, representing the first cause of oncological death [14]. Recent statistics suggest that its worldwide incidence and the associated mortality are on the rise. Breast cancer is a heterogeneous disease divided into different molecular subtypes according to the expression of biological markers: the estrogen receptor (ER), progesterone receptor (PR) and human epidermal growth factor receptor 2 (HER2) [15]. Among the different breast cancer types, triple-negative breast cancer (TNBC), a specific subtype that does not express ER and PR and does not overexpress HER-2, is characterized by high invasiveness, high metastatic potential and poor prognosis [16]. The molecular subtypes may impact prognosis and may influence decisions about the therapeutic management. Conventional approaches, including hormone therapies, have shown insufficient efficacy, especially for the treatment of the TNBC. However, the chemotherapeutic agents are still considered as the first line therapy in aggressive breast cancer, despite their dramatic side effects [17]. The limitations of the current breast cancer therapeutic approaches, mostly linked to the severe toxicity and the onset of frequent resistance phenomena, reflect the need to develop new agents with improved pharmaceutical profiles. One of the most widely used strategies in breast cancer therapy has been the induction of the programmed cell death, namely apoptosis, in tumor cells [18]. Apart from this, signaling pathways and metabolic components involved in the development and progression of breast cancer could represent potential targets for the design and synthesis of new useful compounds in cancer treatments. As well as multiple and specific cellular components, often over-expressed or altered in cancerous cells, such as topoisomerases, proteins cytoskeletal and so on, are important for the discovery of new anticancer drugs

**Figure 1:**
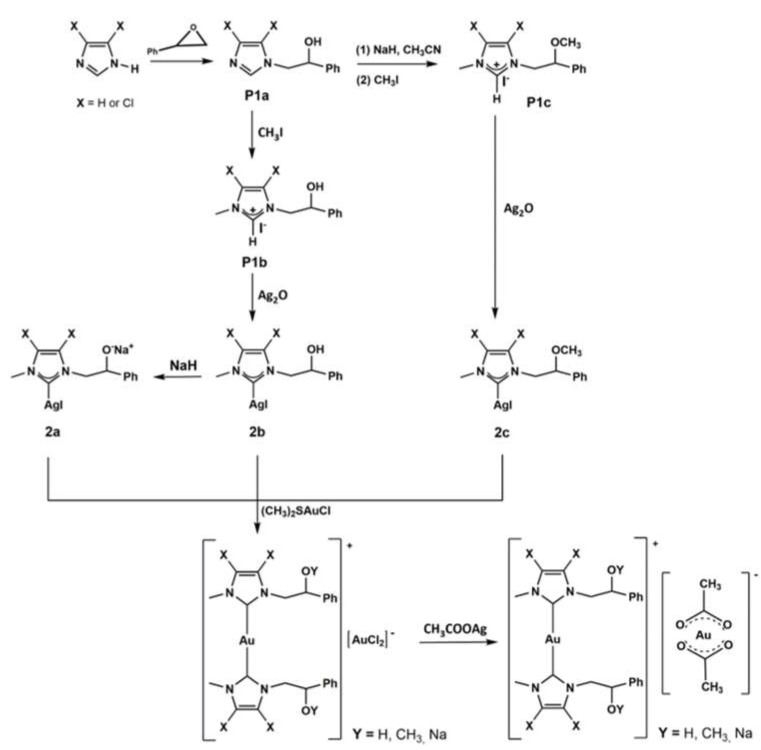
New synthesized Au *N*-heterocyclic (NHC) carbene complexes.

In this scenario, in order to enhance the solubility and the biological activity, we structurally modified some already studied complexes with promising anticancer properties [5,7,13], obtaining new Au-NHC derivatives (Figure 1). 

Among the new gold NHC complexes, compounds **1** and **6** possessed the best anticancer activity towards two breast cancer cell lines (MCF-7 and MDA-MB-231). In vitro and in silico studies demonstrated their capability to inhibit two crucial enzymes involved in DNA metabolism, namely the human topoisomerases I (hTopo I) and II (hTopo II). Furthermore, immunofluorescence and specific in vitro assays allowed us to establish that the compounds **1** and **6** are inhibitors of the actin polymerization, a process that is essential for different cells’ functions. As a result, cancer cells are forced to die through the extrinsic apoptotic mechanism. Moreover, these complexes are able to affect the NF-kB translocation into the nucleus and downregulate the vimentin expression. The ability to interfere with metabolic key-points that are altered in cancer cells and responsible for sustaining the abnormal proliferation represents a valuable feature in the oncologic field.

## 2. Results

### 2.1. Chemistry

**Scheme 1:**
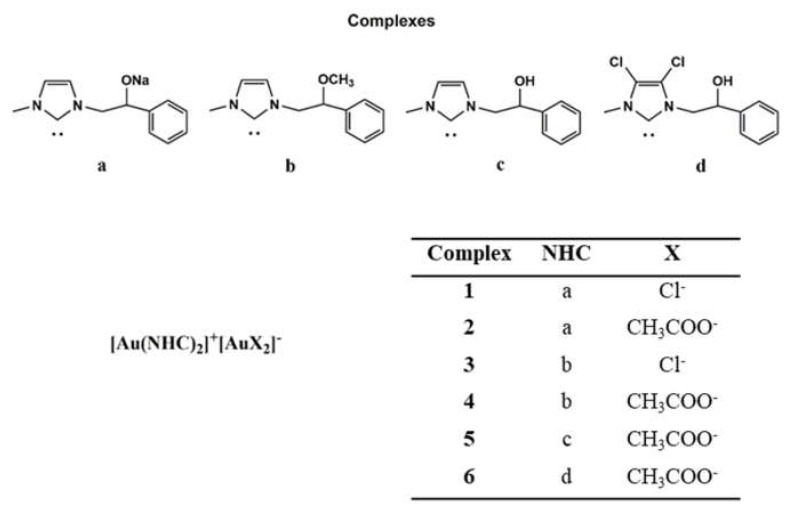
NHC-Au(I) complexes.

The complexes were prepared according to the procedure reported in the literature [19] and shown in Figure 1 and Scheme 1. The NHC-gold(I)-OAc complexes (**2**, **4**, **5**, **6**) were obtained by halogen metathesis reaction, as shown in Scheme 1, starting from the respective NHC-gold(I) chloride complex [20]. 

Imidazole or 4,5-dichloroimidazole was reacted with styrene oxide to give *N*-alkylated products (**P1a**), i.e., *N*-2-hydroxy-2-phenyl-ethyl-imidazole or *N*-2-hydroxy-2-phenyl-ethyl-4,5-dichloroimidazole. **P1a** give rise to proligand **P1c** reacting with sodium hydride in acetonitrile followed by reaction with iodomethane at room temperature for 24 h. Deprotonation of **P1c** proligand by Ag_2_O affords the silver complex **2c**, that by trans metalation reaction with gold(I)-chloro-dimethylsulfide [(SMe_2_)AuCl] produced the light brown NHC–gold complex **3**. 

Gold complex **1** was synthesized by reaction of **P1a** with methyl iodide giving **P1b**. This imidazolium salt reacting with silver oxide produces the carbene NHC able to coordinate the metal giving the complex **2b**. Reaction of **2b** with sodium hydride produces the complex **2a**. Gold complex **1** is achieved by the reaction of silver complex **2a** with gold(I)-chloro-dimethylsulfide [(SMe_2_)AuCl]. 

The gold chloride complexes precursors of **5** and **6** were obtained by reaction of **P1b** with silver oxide, followed by trans metalation with gold(I)-chloro-dimethylsulfide [13,20]. 

All the synthesized products were analyzed by means of ^1^H and ^13^C NMR, mass spectroscopy (ESI or MALDI) and elemental analysis techniques, and the success of the syntheses carried out was confirmed by comparison with the data reported in the literature [13,19,20].

The **2**, **4**, **5**, **6** acetate complexes were obtained in 45%, 55%, 50% and 60% yields, respectively, by reacting the suitably substituted NHC-gold(I) chloride with 1.2 equivalents of silver acetate in dry CH_2_Cl_2_ at 0 °C for 3 h, excluding light. The reaction mixture was filtered over celite and the solvent removed under reduced pressure. NMR spectra of the metal complexes were recorded in DMSO-*d*_6_ or CDCl_3_ at room temperature. The ^1^H and ^13^C NMR spectra show the predictable signals. For all complexes the exchange of the counterion was confirmed by the presence of the three protons attributable to the methyl group in ^1^H NMR and by the two new carbon peaks in the ^13^C NMR spectra. The resonance of protons of methyl group was at around 1.8 ppm, whereas the carbons chemical shifts were observed at around 23 ppm and 175 ppm and attributed to the methyl and carbonyl carbon of the acetate ligand, respectively. As further confirmation of the exchange of the chloride with acetate ligands, it should be noted that for all the complexes a shift of the carbene carbon was detected (Table 1).

These data are in agreement with the hypothesis of Herrmann et al. [21] that suggested a move of carbenic carbon, in the ^13^C NMR, to uphold the chemical shift as the Lewis acidity of the metal center decreases. Of course, other factors, such as metal oxidation state and properties of NCH ligand, contribute to the Lewis acidity of the metal.

For all complexes, the elemental analysis gives the predictable composition in C, H and N, and mass spectroscopy analysis is consistent with [(NHC)_2_Au]^+^ (see experimental part). From literature, a dynamic equilibrium in solution is known between mono and bis carbenic metallic species, as evidenced by the solid-state structure determined by X-ray diffraction of analogous complexes [22]. For all synthesized compounds the logarithm of the partition coefficient (LogP) was determined (see experimental part). The LogP value represents an important molecular characteristic for the action profile of a drug, because it influences both pharmacokinetic and pharmacodynamics. The LogP value of the synthesized complexes is reported in Table 2; it is calculated by evaluating the concentration ratio of each compound in the two phases constituted by the binary immiscible octanol-water mixture. As can be seen, in accordance with expectations, the acetate complexes have a higher solubility in the aqueous phase than chloride complexes, while maintaining a significant lipophilicity. In fact, all the synthesized complexes have a LogP included in the optimal range for the “drug-like” molecules. The range obtained varies from 0.079 to 0.78. Comparing the values of complexes 1 with 2, and of 3 with 4, it was possible to evaluate the better solubility in water of the complexes with acetate compared to those with chloride as counterion.

### 2.2. Biology

#### 2.2.1. Anticancer Activity

The ability of the new series of Au-NHC complexes against three breast cancer cell models, viz. the ER-α positive MCF-7, the highly aggressive and metastatic triple negative MDA-MB-231 cells and the ER-α negative SkBr3, was assayed by the means of the MTT assay. Our outcomes (Table 3) indicated the compound **6** as the most active of the series against the MCF-7 cells, indeed it drastically reduced their growth with an IC_50_ value of 1.2 ± 0.3 μM. A similar activity was found toward the ER-α negative SkBr3 with an IC_50_ value of 2.3 ± 0.9 μM. Conversely, a lesser activity was recorded against the MDA-MB-231 cells (IC_50_ = 16.8 ± 1.2 μM), whereas it was slightly toxic against the normal MCF-10A and Hek-293 cells (IC_50_ = 24.4 ± 0.9 and 32.5 ± 1.1 μM, respectively) even though about 20- and 27-fold less active than against the MCF-7 cells, respectively. Instead, the compounds **3** and **4** were very active against all the breast cancer cells lines but, unfortunately, they also exhibited a high toxicity against the normal MCF-10A and Hek-293 cells. The compound **1** showed a fairly good anticancer activity towards the MDA-MB-231 cells, with an IC_50_ value of 15.8 ± 0.7 μM, but together with a complete lack of cytotoxicity against the normal MCF-10A and Hek-293 cells, up to the concentration of 200 μM. Finally, the compounds **2** and **5** displayed a non-selective behavior towards the cancer and normal breast cell lines, exhibiting similar IC_50_ values. However, both the compounds did not exert any cytotoxicity on the human embryonic kidney epithelial Hek-293 cells (IC_50_ > 200 μM). Thus, we decide to investigate some of the possible intracellular targets of the most promising compounds, discarding those that did not have a suitable cytotoxic profile. The best candidates we chose were the compounds **1** and **6**, which possess the best anticancer activity amongst the series. Moreover, if compared with the two reference molecules used in our assays, *Cis*platin and Latrunculin A (LA), these complexes demonstrated a better anticancer activity with respect to the *Cis*platin on both of the breast cancer cells, and a lesser cytotoxicity on the MCF-10A normal cells than Latrunculin A. 

#### 2.2.2. Compounds **1** and **6** Inhibit Both the Human Topoisomerases I and II

**Figure 2:**
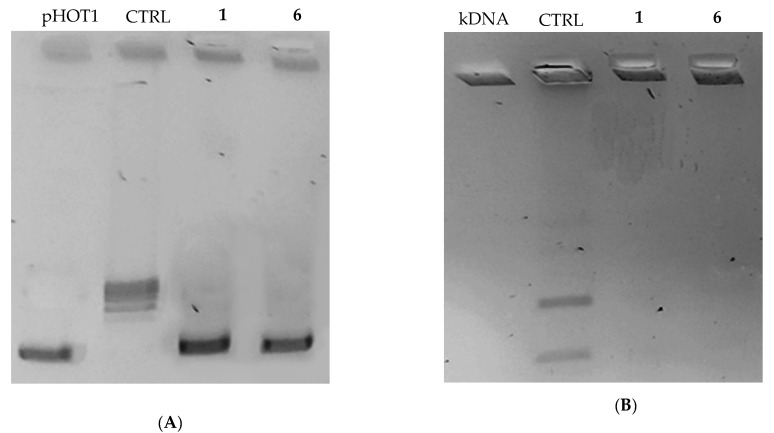
hTopo I and hTopo II assays. (**A**) hTopo I relaxation assay. hTopo I was exposed to DMSO (CTRL) or compounds **1** and **6** at the concentration of 1 μM, then the products of hTopo I reaction were visualized on agarose gel; (**B**) hTopo II decatenation assay. hTopo II was exposed to DMSO (CTRL) or compounds **1** and **6** at the concentration 1 μM. The hTopo II reaction products were visualized on agarose gel. pHOT1, supercoiled plasmid DNA; kDNA, kinetoplast DNA; CTRL, control (DMSO); **1** and **6**, tested compounds.

In the last few years, several new gold complexes holding many biological activities have been reported [23,24,25], as well as the different intracellular targets, viz. kinases, proteases, reductases and, last but not least, topoisomerases [6,26,27]. Following this research field, we intended to study the ability of the new lead compounds **1** and **6** to block these enzymes, strongly implicated in cancer cells’ proliferation and, for this reason, two of the major targets of various studies addressed to find new candidate drugs in the continuous fight against cancer. Human DNA topoisomerases are essentially involved in assuring the right DNA replication in normal cells, thanks to their topological functions but, unfortunately, their action is strictly and mostly required in cells proliferating without any control, as it happens in cancer cells where these enzymes are overexpressed. It is evident that these enzymes represent an attractive target to study for the development of even more specific therapies. To assess the capability of compounds **1** and **6** in inhibiting both the human DNA topoisomerases, we performed an in vitro inhibition assay. As a principal function of the human Topoisomerase I (hTopo I) is the relaxation of a supercoiled DNA, in this assay the hTopo I was exposed to each lead in presence of its substrate and, at the end, the reaction products were analyzed by the agarose gel electrophoresis. Our outcomes indicated that both the compounds were able to totally inhibit the hTopo I supercoil relaxing activity, already at the concentration of 1 μM, as evidenced by a clear band of uncut plasmid DNA at the bottom of the gel (Figure 2, Panel A, compounds **1** and **6**). On the contrary, in the presence of the only vehicle (DMSO) the hTopo I retained its full activity, under the same experimental conditions, as visible in the control lane, where it was possible to observe the presence of multiple bands corresponding to the cut DNA plasmid (Figure 2, Panel A, CTRL). As marker we used the uncut plasmid pHOT1 (Figure 2, Panel A, pHOT1).

The human Topoisomerase II (hTopo II) possesses the ability to catalyze the decatenation of a double-stranded DNA. Thus, the interlocked kinetoplast DNA (kDNA) was used as substrate in the hTopo II inhibition assay, in the presence or absence of the compounds **1** and **6** at the concentration of 1 μM. The reaction products were resolved by gel electrophoresis and, as visible in Figure 2, as well in this case both the compounds totally inhibited the enzyme activity. Indeed, a notable band in the upper of the gel represents the catenated circles of kDNA, unable to enter the agarose gel (Figure 2, Panel B, compounds **1** and **6**). Conversely, in the absence of compounds **1** and **6** (control reaction, with DMSO only), two bands related to the decatenation products were visualized at the bottom of the agarose gel (Figure 2, Panel B, CTRL), indicating the enzyme full activity. Summing up, we evidenced that both the leads possess the ability to block the hTopo I and II activity, which evidently represent two of the targets that are involved in the anticancer activity. 

#### 2.2.3. Compounds **1** and **6** Inhibit the Actin Polymerization

**Figure 3:**
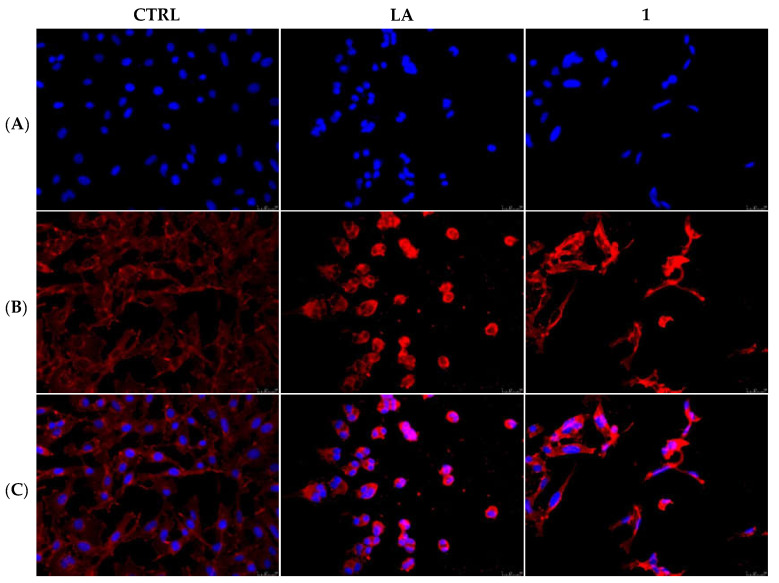
Actin immunofluorescence studies. MDA-MB-231 cells were exposed to compound **1** (used at its IC_50_ value), with 0.1 μM Latrunculin A (LA) or with a vehicle (CTRL) for 24 h. Then the cells were further processed, observed and imaged under the inverted fluorescence microscope at 40x magnification. CTRL cells showed a regular arrangement and organization of the actin cytoskeleton. MDA-MB-231 cells exposed to LA and, as well as those treated with compound **1**, exerted an irregular arrangement and organization of the actin system. Panels (**A**): nuclear stain with DAPI (λ_ex_/λ_em_ = 350/460 nm); Panels (**B**): β-actin (Alexa Fluor^®^ 568; λ_ex_/λ_em_ = 644/665 nm); Panels (**C**): show a merge. Representative fields are shown.

**Figure 4:**
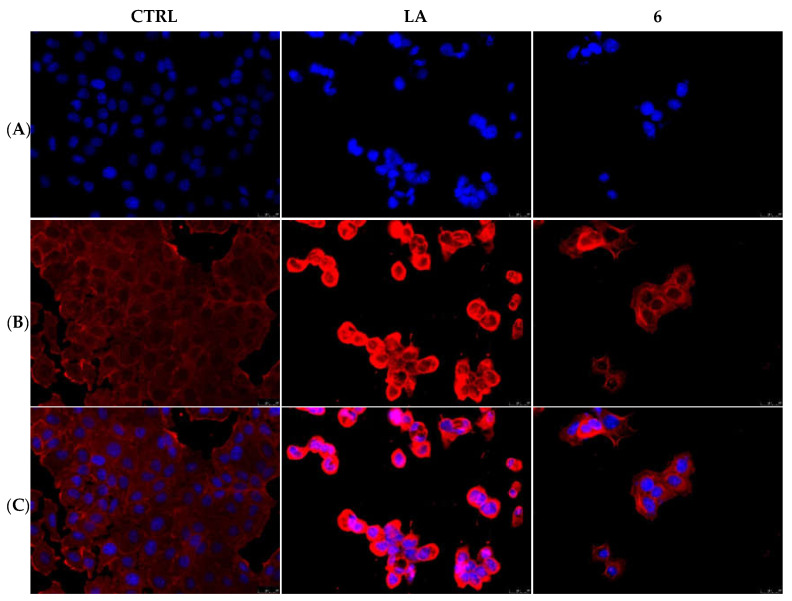
Actin immunofluorescence studies. MCF-7 cells were exposed to compound **6** (used at its IC_50_ value), with 0.1 μM Latrunculin A (LA) or with a vehicle (CTRL) for 24 h. Then the cells were further processed, observed and imaged under the inverted fluorescence microscope at 40× magnification. CTRL cells showed a regular arrangement and organization of the actin cytoskeleton. MCF-7 cells exposed to LA and, as well as those treated with compound **6**, showed an irregular arrangement and organization of the actin system. Panels (**A**): nuclear stain with DAPI (λ_ex_/λ_em_ = 350/460 nm); Panels (**B**): β-actin (Alexa Fluor^®^ 568; λ_ex_/λ_em_ = 644/665 nm); Panels (**C**): show a merge. Representative fields are shown.

The actin cytoskeleton is a structural network involved in several, and probably not yet all discovered, biological functions, such as cells contraction and motility, intracellular vesicle transport, endocytosis and cells’ death. In the last case, the actin filaments organization undergoes dramatic changes accompanying the several apoptosis stages [28]. Our previous studies on gold-NHC complexes [29] revealed a role in disturbing the actin equilibrium, thus with the aim to investigate whether our lead compounds may interfere with the actin system, we performed immunostaining and in vitro direct enzymatic assays. First, MDA-MB-231 and MCF-7 cells were treated for 24 h with compounds **1** and **6** (used at their IC_50_ values), respectively, together with the only vehicle (DMSO, negative control) and LA (positive control) at a concentration of 0.1 µM. Then, cells were processed and observed under a fluorescent microscope, as detailed in the experimental section, and the obtained results indicated that, in the DMSO-treated cells, the actin filaments are thin and regularly organized throughout the cytoplasm (Figure 3 and Figure 4, panels A). Conversely, the LA-treated cells showed a dramatic change in shape, becoming circular, because of the induction of a complete disorganization of the actin system. Indeed, in Figure 3 and Figure 4, panels B, it is possible to notice that actin bundles appeared thicker and brighter with respect to the vehicle-treated cells, and accumulated in dot-like structures. The MDA-MB-231 cells, under the compound **1** treatment, lost their shape, as well, but assumed a threadlike morphology, because of the altered actin bundles organization. Finally, in the MCF-7 cells treated with the compound **6**, the actin bundles appeared thicker and dramatically packed into the cytoplasm and, consequently, the normal cell shape was completely lost (Figure 4, panel B). A parallel experiment has been conducted on MCF-10A and reported in the Supplementary Materials (Figure S1). Thus, we can deduce that both the compounds interfere with the regular organization of the actin system in the breast cancer cells under investigation. 

**Figure 5:**
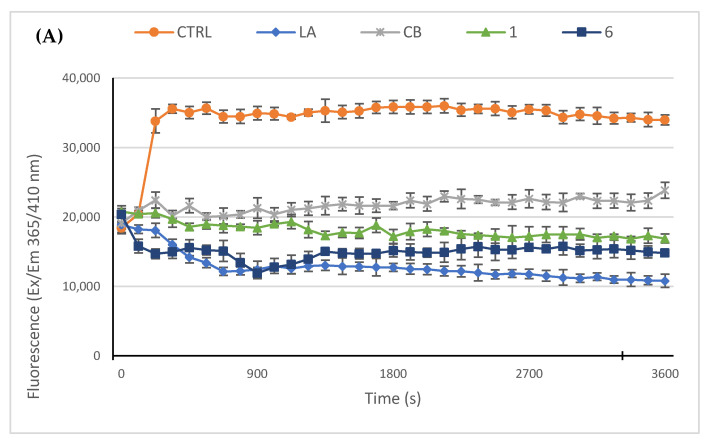

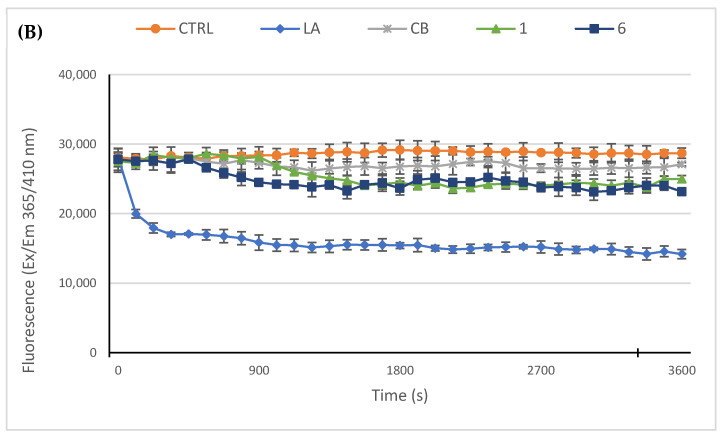
In vitro actin polymerization/depolymerization assay. The effect of compounds **1** and **6** (5 μM) on in vitro actin polymerization and depolymerization was examined. DMSO was used as a negative control. Actin-targeting agents, latrunculin A (LA) and cytochalasin B (CB), both at the concentration of 5 μM, were used as controls. (**A**) In vitro actin polymerization assay: compounds **1** and **6** were incubated with the labeled rabbit muscle actin in order to verify their ability to inhibit the protein polymerization; (**B**) In vitro actin depolymerization assay: after actin polymerization, the compounds **1** or **6** were added to the reaction mixture, in order to determine their ability to act as depolymerizing agents. For both the assays, the assembly of actin filaments was determined by measuring the fluorescence (Ex/Em: 365/410 nm) in kinetic mode for 1 h at room temperature using a microplate reader. The graphics are representative of three different assays.

Next, we used an in vitro actin polymerization/depolymerization assay (based on a fluorescent-labeled purified rabbit actin, whose fluorescence rises during the polymerization reaction) in order to confirm the immunofluorescence results and to investigate if the compounds **1** and **6** could act as actin polymerization mechanism inhibitors and/or accelerate the F-actin depolymerization. As reference molecules, in this assay we employed the LA and cytochalasin B (CB). Indeed, LA is able to bind and sequester G-actin monomers, impeding the polymerization but, as well, promote the subunits dissociation from the ends of assembled filaments with the same final result, which is impeding the F-actin formation. On the contrary, CB inhibits the rate of actin polymerization blocking the actin monomers addition to the “barbed” end of the filaments, without disassembling the prominent actin bundles. In the control reaction (only vehicle), actin monomers polymerize rapidly and normally, as visible in Figure 5, panel A; indeed, the reaction curve increases in about 5 min, reaching a value of about 37.000 RFU (see experimental section for details) and maintaining the plateau until the end of the experiment. Conversely, the two inhibitors used as reference molecules, namely LA and CB at a concentration of 5 µM, impeded the actin polymerization and showed a lack of the initial rapid curve growth, as observed in the control reaction. In particular, LA efficacy seems higher with respect to that of CB under the adopted experimental conditions, indeed the LA curve decreased, until a value of about 12.000 RFU, 14 min after the reaction started, then maintained the same value and ended at a value of about 10.000 RFU. The CB curve, instead, showed a minimal increase in the first 4 min, and then maintained the plateau until it ended almost at the same RFU value of the beginning. Finally, our compounds **1** and **6**, used at the concentration of 5 µM, exhibited both an inhibiting activity higher that CB but lesser than LA. Specifically, the compound **6** curve was lower than that of LA in the first 7 min but, then, reached a final value of about 14.800 RFU, whereas the final value of compound **1** was 17.000 RFU. Next, we performed the depolymerization assay, in order to understand whether our compounds can act as actin depolymerizing agents, as in the case of LA. Thus, we allowed actin to polymerize for one hour, under the same experimental conditions used above, and then we added the molecules to test, at the concentration of 5 µM, and followed the reactions for another hour. As visible in Figure 5, panel B, the LA addition to the polymerized actin induced a net decrease of the curve after about seven minutes, which indicates that the LA triggered the actin depolymerization, until a value of about 16.800 RFU. The initial value was around 28.000 RFU and the curve, after the initial rapid reduction, continues to decrease very slowly until it ends at the value of 14.000 RFU. The control reaction (only vehicle) maintained, instead, nearly the same initial value, indicating that the actin polymerization reached the maximum; the exposure to CB, compounds **1** or **6** did not produce a significant decrease in the curves, indicating that they do not act on the already polymerized actin, as expected for the CB and as outcome for our compounds. Summing up, the obtained results indicate that both the compounds **1** and **6** are actin polymerization inhibitors with a better efficacy than CB, at the same concentrations and under the adopted experimental conditions, and that they do not accelerate actin depolymerization as the LA did.

**Figure 6:**
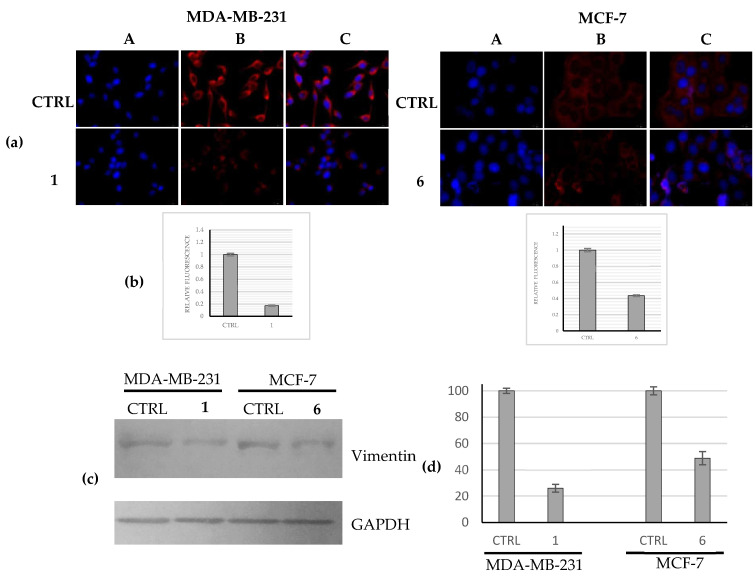
Evaluation of vimentin expression levels. (**a**) Immunofluorescence studies: MDA-MB-231 and MCF-7 cells were treated with compounds **1** and **6**, respectively, (used at their IC_50_ values) or with a vehicle (CTRL) for 24 h. After treatment, the cells were further processed, observed and imaged under the inverted fluorescence microscope at 40× magnification (for more details see Materials and Methods). Compound **1** induced a downregulation of the vimentin expression with respect to the CTRL cells in MDA-MB-231 cells. Panels A: nuclear stain with DAPI (λ_ex_/λ_em_ = 350/460 nm); Panels B: vimentin (Alexa Fluor^®^ 568; λ_ex_/λ_em_ = 644/665 nm); Panels C: show a merge. Images are representative of three separate experiments; (**b**) Fluorescence quantification carried out using ImageJ; *p* < 0.001; (**c**) Western blots analysis: MDA-MB-231 and MCF-7 cells were treated with compounds **1** and **6**, respectively, (used at their IC_50_ values) or with a vehicle (CTRL) for 24 h and then the total protein content was extracted and processed as reported in the Experimental Section. GAPDH: loading normalization. Images are representative of three separate experiments; (**d**) Western blots quantification carried out using ImageJ; *p* < 0.001.

Another important component of cytoskeleton is vimentin, an intermediate filament involved in vital cellular functions and associated as well with an increased capability to metastasize and a poor prognosis in multiple cancer types if overexpressed [30,31]. In order to determine whether the most active complexes (**1** and **6**) were able to regulate the vimentin expression in the adopted breast cancer cells, we used immunofluorescence analyses. Our outcomes indicated that compound **1** strongly reduced the expression of vimentin, at the protein level, in the highly metastatic MDA-MB-231 if compared with the control cells (Figure 6a, MDA-MB-231, panels B, **1** and CTRL). The fluorescence quantification revealed a reduction of about six-fold of the vimentin expression with respect to the control (Figure 6b, MDA-MB-231). Similarly, the complex **6** produced a two-fold reduction of vimentin expression level in MCF-7 cells with respect to the control cells, treated with the vehicle only (Figure 6a,b, MCF-7, **6** and CTRL). A control experiment on the normal counterpart (namely, MCF-10A) has been performed (Supplementary Materials, Figure S2). These findings suggest that the downregulation of vimentin expression could support the observed interference with the actin network. Similar results were obtained by using WB analysis (Figure 6c,d).

#### 2.2.4. Docking Studies

**Figure 7:**
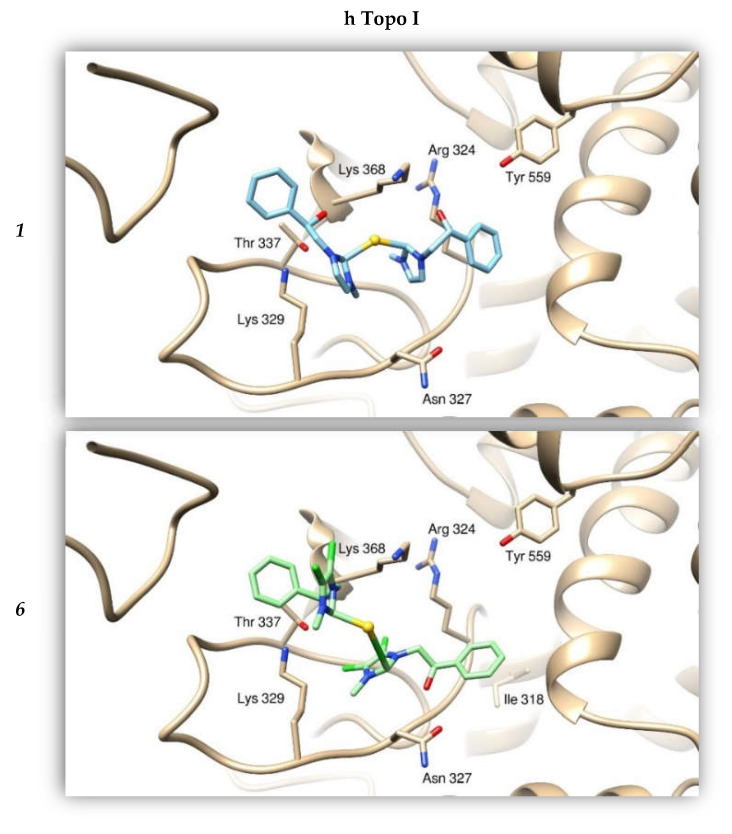

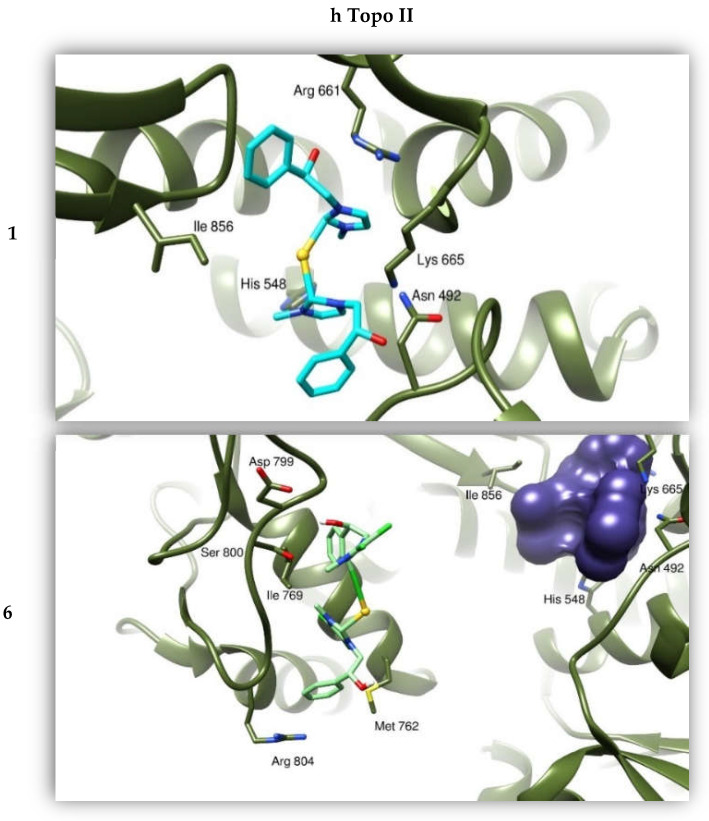
Binding modes of compounds **1** and **6** with hTopo I and hTopo II. The proteins are depicted as tanned and olive-green ribbons, respectively. Compound **1** is drawn as light blue sticks, while compound **6** as green sticks. As reference, the violet bubble indicates the position of compound **1** with respect to compound **6** in binding hTopo II.

We calculated the affinities between compounds **1** and **6** and the protein targets by performing a “blind-docking approach” (no “a priori” information about the binding site was provided to the system) for all our docking simulations. This way, we determined the most promising candidate aiming to further improve the atomic structure of our compounds. We analyzed the binding modes of the compound to the two Topoisomerases, and calculated the binding affinities using the program AutoDock (this program calculates a binding affinity constant K_i_ on the basis of the knowledge of the binding Energy between the two entities, according to the expression *Ki* = exp (deltaG/(R*T)). Moreover, we took into consideration the clustering of the results from the simulations, as discussed in previous works [32]. The resulting binding mode was visually examined to evaluate the quality of the protein: ligand interactions. Our compounds were able to bind with a high energy both the hTopo I and II (see Table 4), forming several hydrogen and hydrophobic interactions, positioning within a channel where DNA is normally bound. In particular, compound **1** interacts with hTopo I by forming hydrogen bonds with residues Arg324, Thr337 and Lys368 and hydrophobic interactions with His203, Ala334 and Tyr559. The Au atom is coordinated to the ammine group of Lys329. The same compound **1** forms hydrogen bonds with the hTopo II residues Asn492, Arg661 and Lys 665, this one is also involved in gold coordination. The hTopo II hydrophobic residues Leu491, Phe653 and Ile856 contribute to the stabilization of this interaction. The compound **6** binds the hTopo II differently from compound **1**, forming H-bonds with Lys798, Ser800 and Arg804, and hydrophobic interactions with Met762, Met766 and Ile769. In this last case, the Au atom does not seem to contribute to the binding (Figure 7). 

**Figure 8:**
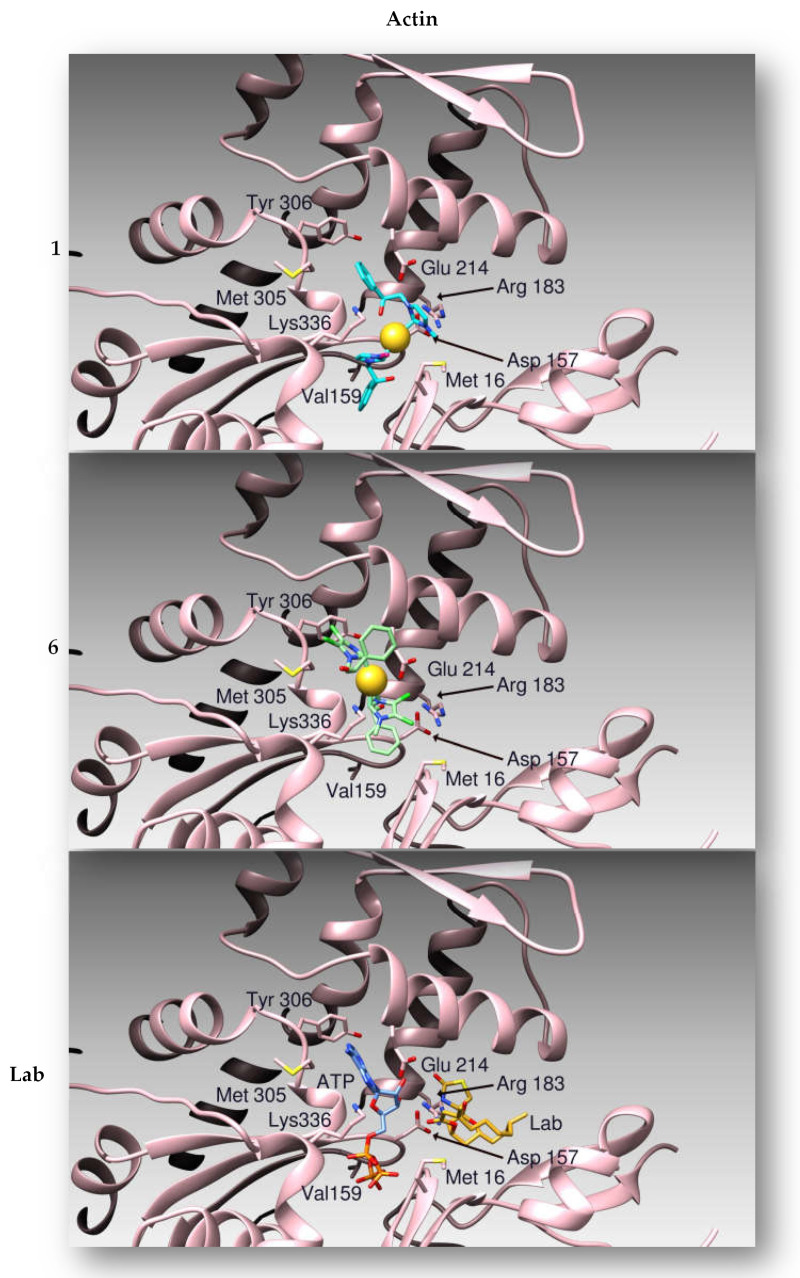
Ribbon representation of the binding modes of compounds **1**, **6** and Latrunculin B (Lab) to Actin. The proteins are drawn as pink ribbons. Compound **1** is drawn as light blue sticks, while compound **6** as green sticks. The binding pose of Latrunculin B (Lab), as determined by X-ray crystallography, is reported as gold sticks.

The same protocol was adopted using the atomic coordinates of the protein Actin as a target. By molecular docking simulations we evaluated the binding modes and calculated the affinities between **1** and **6** to the target protein [33]. Compounds **1** and **6** share the same Actin binding site with Latrunculin B (Figure 8), as previously determined by X-ray crystallography [33], where **1** forms hydrogen bonds with protein residues Asp157 and Arg183, while the gold atom is coordinated by Glu214. The binding site is stabilized by hydrophobic interactions with residues Met16, Met305 and Tyr306. On the other side, compound **6** forms hydrogen bonds with protein amino acids Asp157, Thr303 and the peptidic nitrogen atom of Gly182. Moreover, Glu 214 interacts thanks to a halogen bond with a chlorine atom of the ligand, which is further stabilized by hydrophobic interactions with Met16, Met305 and Tyr306. The Au atom is coordinated to Lys336. 

#### 2.2.5. Compounds **1** and **6** Were Able to Trigger Apoptosis in Breast Cancer Cells

**Figure 9:**
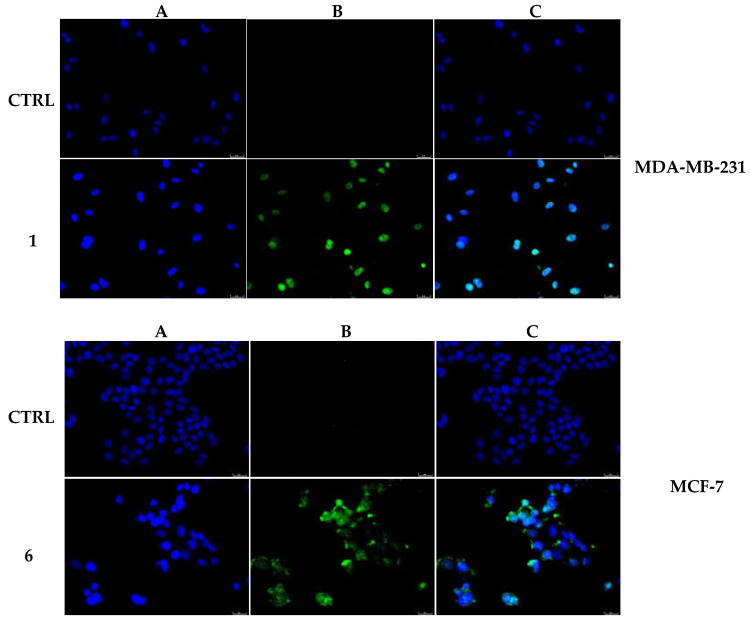
TUNEL assay. MDA-MB-231 and MCF-7 breast cancer cells were treated with compounds **1** and **6**, respectively, at the concentration equal to their IC_50_ or with vehicle (CTRL) for 24 h. Then they were exposed to the TdT enzyme and visualized under a fluorescence microscope (20× magnification). The green fluorescence indicates nuclei of cells undergoing apoptosis. Panels (**A**), DAPI λ_ex/em_ 350 nm/460 nm. Panels (**B**), CF^TM^488 A λ_ex/em_ 490 nm/515 nm. Panels C show the overlay channel.

Topoisomerases I and II are ubiquitous enzymes involved in the DNA duplication and represent important anticancer targets because their inhibition blocks cancer cells’ proliferation and are classified as the most efficient inducers of DNA damage that leads to cancer cell death by apoptosis [5,6,34,35]. Moreover, agents targeting the actin cytoskeleton have been proved to mediate apoptosis [28,36,37]. To verify the capability of both compounds **1** and **6** to trigger apoptosis, a TUNEL assay was performed. Figure 9 (MDA-MB-231) shows that compound **1** induced apoptosis in MDA-MB-231 breast cancer cells. Indeed, it is possible to notice the presence of a remarkable green fluorescence, indicating DNA fragmentation. This fluorescence is absent in the control cells, treated with DMSO only, indicating the lack of a DNA damage. The same results were obtained for the compound **6**-treated MCF-7 cells, as visible in Figure 9 (MCF-7). MCF-10A cells exposed to both the compounds showed no apoptosis (Supplementary Materials, Figure S3). These data confirm that the observed decrease in breast cancer cells viability is due to the activation of the apoptotic pathway, which is the consequence of the blockade of both of the types of hTopo and the inhibition of actin polymerization.

**Figure 10:**
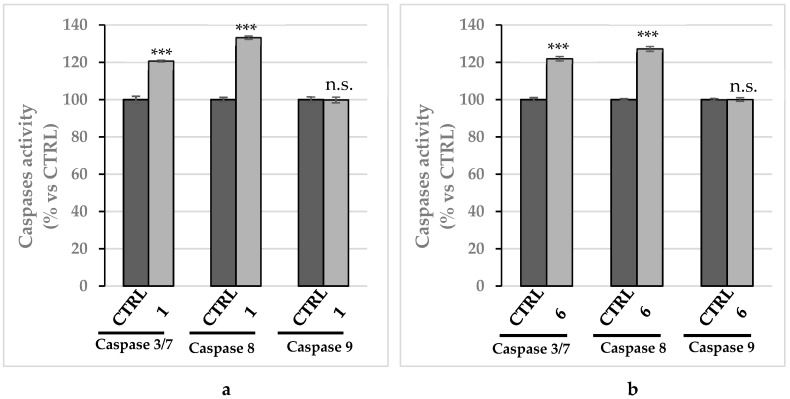
Caspases’ activity. Increase of caspases 3/7 and 8 activity levels, reported as percentage over the vehicle-treated cells used as control, following treatment of MDA-MB-231 cells with compound **1** (**a**) and MCF-7 cells with compound **6** (**b**). Both the compounds were used at their IC_50_ values and incubated for 24 h. The data are representative of three independent experiments. *** *p* < 0.001 and n.s. not significant; treated vs. CTRL.

Next, in order to determine which apoptotic pathway was involved for the observed cell death induced by compounds **1** and **6**, we evaluated the activity of the initiator caspase-8 and -9 and the executioner caspase-3 and -7, by using a luminescent assay. A significant rise in caspase-8 activity was recorded in MDA-MB-231 and MCF-7 cells incubated with compounds **1** and **6**, respectively, used at their IC_50_ values for 24 h, whereas no increase of caspase-9 activity was evinced (Figure 10a,b). Moreover, we detected a clear increase of caspase-3 and -7 activity in both the breast cancer cells treated with compounds **1** and **6** (Figure 10a,b), which it is known are cleaved and activated by the initiator caspase-8. A parallel experiment, used as control, has been performed on MCF-10A and reported in the Supplementary Materials (Figure S4). These observations indicate that the exposure of the breast cancer cells to compounds **1** and **6** trigger the extrinsic apoptotic pathway. 

**Figure 11:**
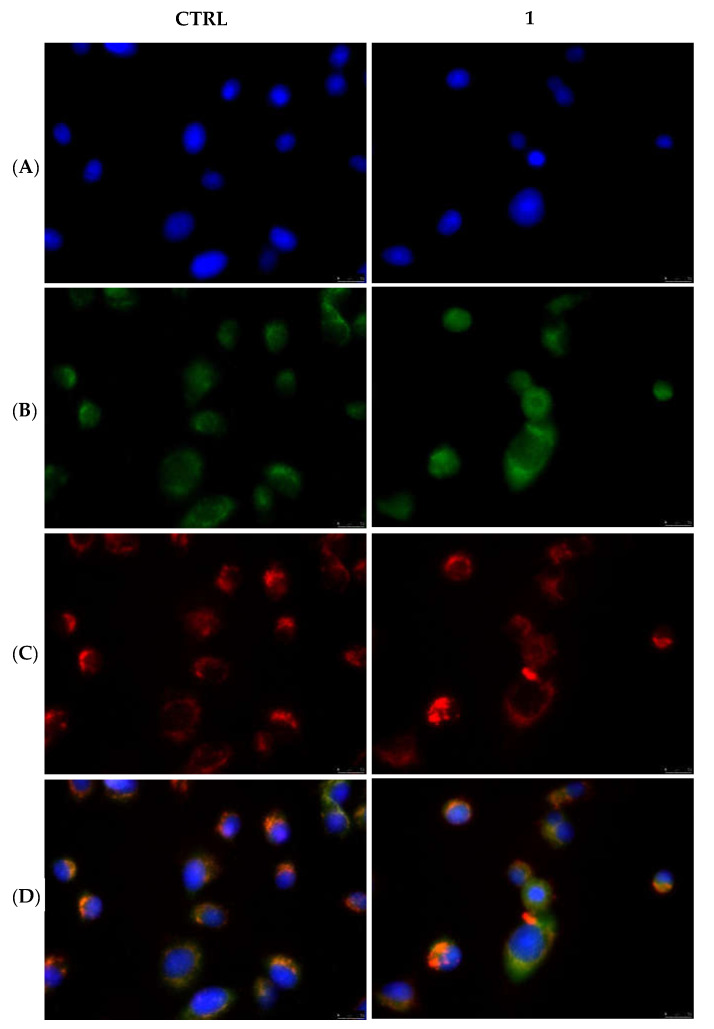
Mitochondria staining and cytochrome c detection in MDA-MB-231 cells. In the CTRL cells as well as in the cells treated with compound **1** at its IC50 value for 24 h, cytochrome c is localized within mitochondria. Panels (**A**), DAPI λ_ex/em_ = 350/460 nm; Panels (**B**), Alexa Fluor CF 488 λ_ex/em_ = 490/515 nm; Panels (**C**), MitoTracker Deep Red FM probe λ_ex/em_ = 644/665 nm; Panels (**D**), overlay channels. Images were acquired at 40× magnification and representative fields are shown.

**Figure 12:**
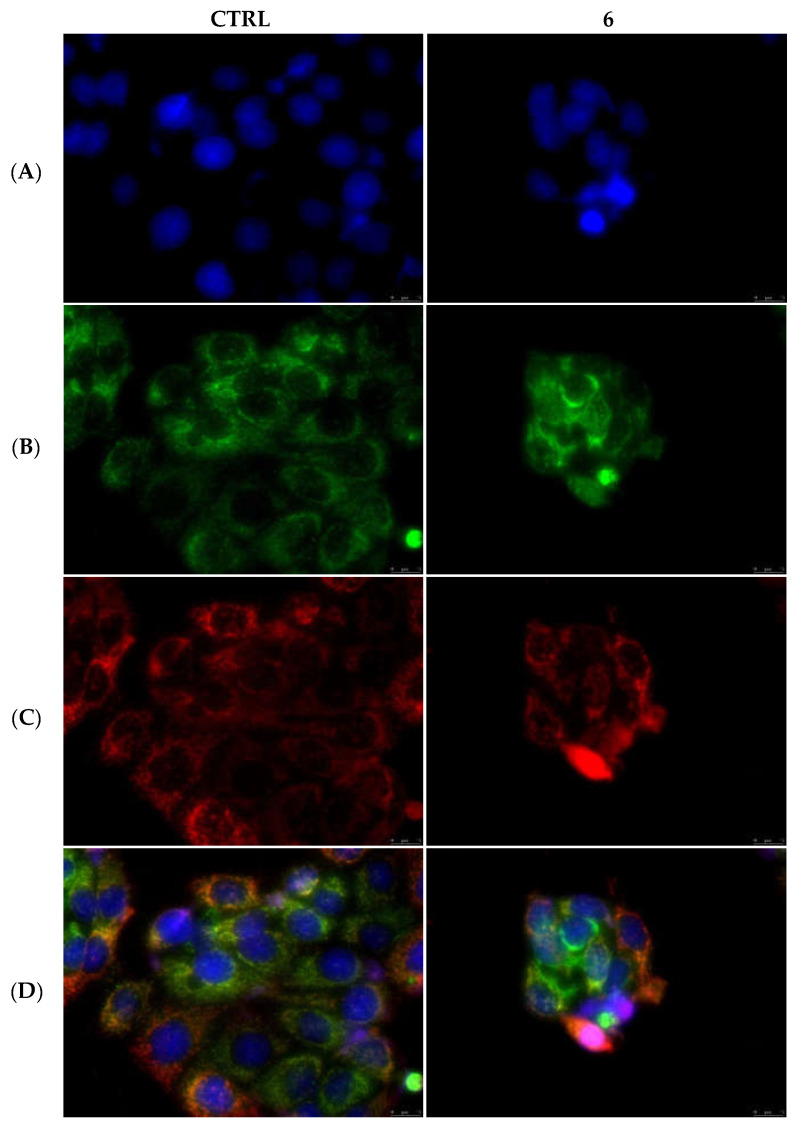
Mitochondria staining and cytochrome c detection in MCF-7 cells. In the CTRL cells as well as in the cells treated with compound **6** at its IC50 value for 24 h, cytochrome c is localized within mitochondria. Panels (**A**), DAPI λ_ex/em_ = 350/460 nm; Panels (**B**), Alexa Fluor CF 488 λ_ex/em_ = 490/515 nm; Panels (**C**), MitoTracker Deep Red FM probe λ_ex/em_ = 644/665 nm; Panels (**D**), overlay channels. Images were acquired at 40× magnification and representative fields are shown.

Differently from the extrinsic apoptotic pathway, one of the hallmarks of the intrinsic pathway is the permeabilization of the outer mitochondrial membrane and the consequent release of the cytochrome c into the cytosol [38]. Again, by the means of immunofluorescence studies, we observed that in the MDA-MB-231 and MCF-7 cells treated with the vehicle only and, as well, in MDA-MB-231 cells treated with compound **1** (Figure 11) and MCF-7 cells treated with compound **6** (Figure 12), the cytochrome c seems to still reside in the mitochondrial network, as visible by the perfect overlay (Panels D) of the red fluorescence associated with the mitochondrial probe (MitoTracker Deep Red FM) (Panels C) and the cytochrome c green fluorescence (Panels B). The nuclear counterstaining was already shown (DAPI, Panels A). A parallel experiment on MCF-10A, used as CTRL, has been performed and reported in the Supplementary Materials (Figure S5). Taken together, our data indicate that the extrinsic pathway is involved in the anticancer activity of our lead complexes. 

**Figure 13:**
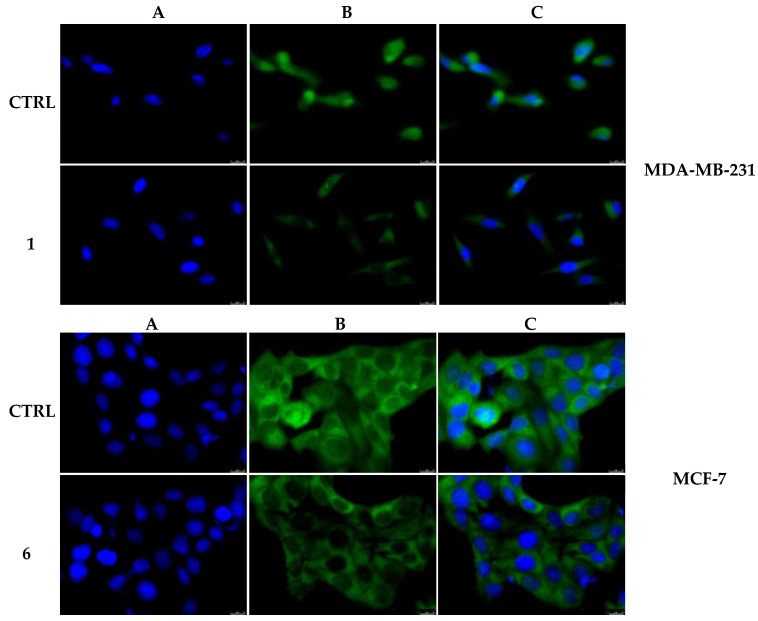
NF-κB activation in MDA-MB-231 and MCF-7 cells after treatment with compounds **1** and **6**, respectively. In the vehicle-treated cells, NF-κB is localized within the cytoplasm and nucleus, while the treatment of the breast cancer cells with compounds **1** and **6** for 24 h (used at their IC_50_ values) reduced the NF-κB presence in the nucleus. Panels A, nuclear stain with DAPI (λ_ex/em_ = 350/460 nm); Panels B, Alexa Fluor CF 488 (λ_ex/em_ = 490/515 nm); Panels C, overlay channels. Images were acquired at 40× magnification and representative fields are shown.

Considering that our complexes **1** and **6** induced apoptosis, increasing the caspases 3/7 and 8 activities without causing the migration of cytochrome c into the cytosol, we investigated the status of NF-κB in our cell models under exposure to our complexes. As visible in Figure 13, the complexes **1** and **6** reduced the translocation of NF-κB from the cytoplasm to the nucleus, in MDA-MB-231 and MCF-7 cells, respectively. Indeed, in the complex **1**-treated MDA-MB-231 cells, the green fluorescence (Figure 13, MDA-MB-231, Panel B, **1**) is predominantly present in the cytoplasm, (Figure 13, MDA-MB-231, panel C, **1**), indicating that the migration of NF-κB from cytosol to the nucleus was diminished. On the contrary, in the vehicle-treated MDA-MB-231 cells (Figure 13, MDA-MB-231, panel B, CTRL), the green fluorescence related to NF-κB is localized in both the cytoplasm and nucleus (overlapping with the blue fluorescence, Figure 13, MDA-MB-231, panel C), where it acts as transcriptional factor.

The same results were obtained in MCF-7 cells treated with the complex **6**, where the green fluorescence in the cell nucleus is clearly lesser (Figure 13, MCF-7, panel B, **6**) than the vehicle-treated cells (Figure 13, MCF-7, panel B, CTRL). A parallel experiment on MCF-10A, used as CTRL, has been performed and reported in the Supplementary Materials (Figure S6).

In conclusion, both the complexes **1** and **6** were able to cause the inhibition of NF-κB translocation from the cytoplasm to the nucleus, where it is transcriptionally active. This evidence confirms the hypothesis of the observed apoptosis triggered by the complexes **1** and **6** and suggests, as well, that the observed diminution in vimentin expression could be due to the inhibition of NF-kB transcriptional activity.

## 3. Discussion

Since the discovery of *Cis*platin, which greatly influenced the antitumor clinical practice, several other platinum-based metallodrugs have been designed and proved to possess a great effectiveness against several tumors [39,40]. However, these molecules exhibited, as well, a number of disadvantages and undesired effects in treated patients [41,42]. These reasons prompted many researchers to explore and design other metals’ complexes, in the hope of producing the most active and selective antitumor drugs [43]. Amongst the metals, the coinage ones, particularly Ag and Au, represented a milestone in medicinal chemistry, given the enthusiastic results obtained from many studies [5,6,7,13,29,44,45,46,47]. Indeed, several complexes exhibited a better anticancer activity and lesser harmful properties than other transition metals against the normal cells. Most importantly, some Au-complexes have been found to be very active against different human diseases, such as rheumatoid arthritis or AIDS [48]. A noteworthy aspect in the design of anticancer metal complexes is their stability under physiological conditions, for better transport and delivery to the cancer cells, which has been successfully achieved using the *N*-heterocyclic carbenes (NHCs) as ligands [49]. Last, but not least, NHCs have been revealed not only as ideal scaffolds less toxic than others and easier to functionalize, but also as effective multitarget agents, able to interfere with relevant and different biomolecules involved in cancer onset and progression [50]. Thus, with the aim to further improve the chemical and biological properties of NHC gold complexes, we designed and synthesized new [NHC-Au(I)]Cl and [NHC-Au(I)]-OAc complexes, with a green approach, and tested their cytotoxic activities against three models of breast cancer cells, namely MCF-7, MDA-MB-231 and SkBr3, and the normal counterpart, MCF-10A cells. We also evaluated the cytotoxicity of our complexes in another normal cell line, the human embryonic kidney epithelial Hek-293 cells, considering that many metal complexes show a certain renal toxicity. This screening indicated the complexes **1** and **6** as the most promising candidates with a better cytotoxic profile, active mostly toward the MDA-MB-231 and MCF-7 cancer cells, respectively. These results come together with a completely lack of cytotoxicity of the complex **1**, and a slight cytotoxicity of complex **6**, against the normal MCF-10A cells. However, it should be highlighted that both the complexes exhibited an improved cytotoxic profile with respect to the reference molecule *Cis*platin, which resulted in less activity against the cancer cells adopted and more cytotoxicity against the normal cells. Fortunately, both complexes **1** and **6** did not exert any cytotoxicity on Hek-293 cells, at least under the adopted experimental conditions. Contrarily, the reference molecules *Cis*platin and Latrunculin A drastically reduced the Hek-293 cells’ viability. Finally, it should be considered that both the complexes exerted a good anticancer activity as well against the SkBr-3 cell line, even to a lesser extent with respect to the above-mentioned breast cancer cells. Several parameters may influence the biological activity of our complexes, such as the different chemical structure, solubility, the ability to pass the cell membranes, the presence of multidrug resistance systems differently expressed in cancer cells, and so on. These variables could explain the diverse IC_50_ values recorded. Thus, we focused our attention on the study of some of the biological targets possibly involved in the revealed anticancer activity. Two of these are represented by the human topoisomerases I and II, which are amongst the most studied targets for tailored cancer therapies [5,51,52]. Using an in vitro assay, we assessed that the leads complexes possessed the ability to inhibit the activity of both the enzymes at a concentration of 1 μM. This is an interesting feature because several studies indicated that the overexpression and the risen activity of both the enzymes are primarily involved in cancer onset, progression, and resistance to the clinically used drugs and may be useful for the diagnosis treatment and prognosis [53,54,55]. These outcomes have been confirmed by the docking simulations, which suggested that both the complexes bind the enzymes with a high energy and form hydrogen and hydrophobic interactions that allow the positioning into a channel where the DNA is normally allocated. Our well-established experience in the study of the multiple biological activities of Au- and Ag-NHC complexes pushed us to investigate whether these new complexes could act as well on cell cytoskeleton, since preliminary observations of cancer cells exposed to the complexes evidenced dramatic morphology changes. Nowadays, it is ascertained that cell cytoskeleton plays a pivotal role in cancer, mostly in the metastatization process, a major cause of death in cancer [56]. The filamentous actin (F-actin) is an important structural cytoskeletal component and its turnover is vital for the normal cells’ functions [57]. Normally, monomeric actin (G-actin) polymerizes in a head-to-tail manner forming helical actin filaments (F-actin) and both the forms are in equilibrium, strictly regulated by different actin-binding proteins [58]. The actin system is implicated in several cellular processes such as exo- and endocytosis, the organelle distribution and movements, cell division, and so on [59]. In cancer cells, the actin cytoskeleton is substantially modified, as well as the associated regulatory proteins. These changes are dramatically involved in the cancer cells’ abnormal growth, their ability to adhere to tissues and to metastasize [60]. Several substances have been proved to alter the intracellular actin organization, representing an attractive target in cancer chemotherapy. Amongst them, cytochalasins and latrunculins are the most used molecules able to permeate the cell membrane and perturb the actin system. In particular, cytochalasin B (CB) reduces the actin polymerization rate inhibiting the actin monomer addition to the “barbed” end of the filaments, whereas latrunculin A (LA) sequesters actin monomers (G-actin) avoiding the actin polymerization and increases, as well, the rate of actin depolymerization [61]. Thus, we focused our attention on actin, as its role in sustaining cancer progression is not yet fairly studied and known. First, by means of immunofluorescence studies we revealed that the exposure of breast cancer cells to the lead complexes induced dramatic changes in the actin organization. In particular, the exposure of MCF-7 and MDA-MB-231 cells to the compounds **6** and **1**, respectively, induced thicker and brighter actin bundles, which are packed into the cytoplasm and not evenly distributed. MCF-7 cells become round, whereas MDA-MB-231 cells assume a threadlike shape, as happens under LA treatment, a well-known inhibitor of actin polymerization. Since LA can inhibit the actin polymerization reaction by sequestering G-actin monomers and, as well, accelerate the subunits dissociation, we wondered if our leads might act in a similar fashion. In order to elucidate this aspect, we performed the in vitro actin polymerization and depolymerization assays, using as reference molecules LA (able to inhibit the polymerization and promote the depolymerization reactions) and CB, which is a polymerization inhibitor. Our outcomes clearly indicated that both the complexes possess the ability to block the actin polymerization reaction, without interfering with the actin depolymerization, behaving in the same way as CB but with a higher efficacy, under the adopted experimental conditions. As well in this case, the in silico studies revealed that both the complexes share the same actin binding site of Latrunculin B and that the gold of the complexes is involved in the interactions with the actin Glu214 (complex **1**) or Lys336 (complex **6**). 

Another important cytoskeleton constituent is represented by vimentin, an intermediate filament involved in a wide spectrum of basic cellular functions, as well as in several diseases and pathological conditions, including high aggressive cancers [30,31], thus it became an important target for the anti-metastasis strategy [62,63]. Indeed, vimentin is strictly connected with tubulin and actin network, participating to cells escape from the primary tumor site [64]. Since elevated vimentin expression is associated with metastasis and unfavorable prognosis in multiple cancer types [31], we aimed to investigate the vimentin expression in our cellular contexts. 

Effectively, the exposure of cancer cells to our complexes evidently reduced the intracellular expression of vimentin with respect to the vehicle-treated cells, with a higher effect produced by complex **1**, able to reduce the protein expression by almost 6 times, whereas the complex **2** exerted a lower effect (two times lower vs. CTRL). Similar results were obtained using WB analyses conducted on the protein extract from cells treated with the proper complex or vehicle. These outcomes strengthened the evidence that these complexes exert a strong action regulating these members of cell cytoskeleton. We also performed a scratch assay and evaluated the expression levels of two proteins involved in ETM transition (namely, E-cadherin and N-cadherin) by immunofluorescence in the adopted cell model. However, at least in our experimental conditions, the complexes under analysis do not seem to regulate cadherins expression, nor arrest the cell migration (data not shown).

Finally, we performed a TUNEL assay in order to verify that the interference with the topoisomerases and cytoskeleton functions could lead to cancer cell death by apoptosis. As expected, the green nuclear fluorescence in both the breast cancer cell lines used in our assays, after the exposure to the complexes, indicate a DNA damage and that apoptosis was taking place. The programmed cell death is generally characterized by distinct morphological features and biochemical mechanisms. Among the effector molecules in apoptosis, proteolytic enzymes such as caspases, play a central role in coordinating the biochemical events that arise during this process [65]. Caspases have been traditionally divided in initiator (caspase-8 and -9) or executioner (caspase-3 and -7) according to their mechanism and the activation of the extrinsic (receptor) or intrinsic (mitochondrial) apoptotic pathway, which is highly dependent on the type of caspases involved [38,66]. The extrinsic apoptotic pathway is activated by the binding of a ligand to a death receptor and involves the activation of caspase-8 that, in turn, cleaves and activate the executioner caspases (-3 and -7) [67]. Instead, the intrinsic pathway is basically activated by different cellular stresses that cause the cytochrome c release from the mitochondria leading to the activation of caspase-9, responsible of the cleavage and activation of the executioner caspases [68]. Literature data reported that different metal complexes may accumulate in the mitochondrial network and induce the intrinsic apoptosis pathway [5,7,65,69]. However, our results suggested the activation of the extrinsic pathway under complexes **1** and **6** exposure given the involvement of caspase-8, an effect that has been already reported for other NHC complexes [70]. One of the hallmark of the intrinsic apoptotic pathway is the permeabilization of the outer mitochondrial membrane and the consequent release of the cytochrome c into the cytosol [38]. In our case, no translocation of cytochrome c was evidenced by our immunofluorescence studies, indicating that the intrinsic pathway is not occurring. Several studies proved that actin cytoskeleton may play a role in triggering both the extrinsic and intrinsic pathways; this depends on different factors or the interplay between various intracellular targets, including the direct interaction between actin and CD95 or FasL, a major ligand of the extrinsic apoptosis pathway [38]. It should be also noted that even the anticancer therapies based on the DNA damaging agents may induce both the apoptotic pathways. For instance, an important role in activating the extrinsic pathway involving both the topoisomerases’ forms and tubulin inhibition was reported [34]. Since we demonstrated that our leads play an inhibitor role against the hTopo I and II and actin polymerization, it is plausible that the effects on these targets are primary (in terms of time or selectivity) with respect to other targets, which deserve future further studies, and that the extrinsic pathways is promoted in the considered cell models.

Lastly, it is well established that the nuclear factor κB (NF-κB), which includes a family of transcription factors implicated in the regulation of several biological pathways, plays a recognized role in the regulation of immune responses and inflammation [71,72]. However, rising evidence sustains its role in cancer since it regulates genes’ expression involved in cancer progression and apoptosis [73,74]. In cancer cells, several molecular modifications may produce an impaired regulation of NF-κB activation, leading to an upregulation or downregulation of gene expression controlled by NF-κB. In most types of cells, the NF-κB dimers are predominantly cytoplasmic but after activation NF-κB migrates into the nucleus where it becomes transcriptionally active [75]. On the other hand, a pro-apoptotic role of NF-κB was proposed in specific cell contexts, where it seems to exert a dual function, either as an inhibitor or an activator of apoptotic cell death [76], and is implicated, as well, in the regulation of the CD95/FasL transcription [77], one of the principal actors in the extrinsic apoptotic pathway. In our case, the complexes **1** and **6** reduced the translocation of NF-kB into the nucleus of MDA-MB-231 and MCF-7 cells, respectively, being prevalently located in the cytoplasm, whereas in the vehicle-treated cells NF-kB exhibited both the nuclear and cytosolic localizations. These results suggested that, in the considered cellular context, the reduced translocation of NF-kB into the nucleus is linked with the observed extrinsic apoptotic pathway and the lower vimentin levels detected under treatment. 

In conclusion, our studies about the new leads reinforce the already established concept of the multitarget ability of the Au-NHC complexes and bring new insights onto another important target in the medicinal chemistry and oncology fields, i.e., the actin, which is strictly connected with tubulin, playing together a key role in controlling cell shape and division. 

## 4. Materials and Methods

### 4.1. Chemistry

All reactions were performed under N_2_ atmosphere, using standard Schlenk and glove-box techniques. All reactions involving silver compounds were carried out excluding light. Ag_2_O, silver acetate and dimethylsulfide-gold(I) chloride were purchased from commercial suppliers [Sigma Aldrich (St. Louis, MO, USA) and TCI Chemicals (Zwijndrecht, Belgium)] and were used as received. The solvents were deoxygenated and dried under nitrogen atmosphere by heating at reflux over suitable drying agents. NMR deuterated solvents were degassed under a nitrogen flow and were stored in the dark, over activated 4 Å molecular sieves. NMR spectra were recorded at 298 K on a Bruker AVANCE 400 spectrometer (400 MHz for ^1^H; 100 MHz for ^13^C) and a Bruker AM 300 spectrometer (300 MHz for ^1^H; 75 MHz for ^13^C). Chemical shifts are given in parts per million (ppm) relative to SiMe_4_ (^1^H, ^13^C) and they were referenced to the residual solvent signals (DMSO-*d*_6_ ^1^H *δ*_H_ = 2.50, ^13^C *δ*_C_ = 39.52; CD_2_Cl_2_: *δ*_H_ = 5.32, ^13^C *δ*_C_ = 53.84) Multiplicities are abbreviated as follows: singlet (s); doublet (d); triplet (t); multiplet (m); broad (br) and overlapped (o). Elemental analyses for C, H and N were recorded with a Thermo-Finnigan Flash EA 1112, and were performed according to standard microanalytical procedures. 

ESI-MS spectra were obtained by using a Waters Quattro Micro triple quadrupole mass spectrometer equipped with an electrospray ion source. MALDI-MS: mass spectra were acquired using a Bruker SolariX XRFourier transform ion cyclotron resonance mass spectrometer (Bruker Daltonik GmbH, Bremen, Germany) equipped with a 7 T refrigerated actively shielded superconducting magnet (Bruker Biospin, Wissembourg, France). The samples were ionized in positive ion mode using the MALDI ion source (Bruker Dalton GmbH, Bremen, Germany). The mass range was set to *m*/*z* 200–3000. The laser power was 28% and 22 laser shots were used for each scan. The mass spectra were calibrated externally using a mix of peptide clusters in MALDI ionization positive ion mode. A linear calibration was applied. To improve the mass accuracy, the sample spectra were recalibrated internally by matrix ionization (2,5-dihydroxybenzoic acid).

Analysis UV/vis spectroscopy have been using a Spectrometer UV-2401 PC (Shimadzu, Japan). The tests were performed using rectangular plates with an exposed area of about 3 cm^2^ and 1 cm of the light path. Final values were shown as the mean of three analyses.

#### General Procedure for the Synthesis of Gold Acetate Complexes

The gold(I) complexes **1** and **3** were prepared according to procedure reported in ref [19]. The acetate gold(I) complexes **2**, **4**, **5**, **6** were obtained by ligand exchange by respective gold(I) chloride, following a slightly modified strategy published in literature [78].

Gold(I) chloride complex (0.10 mmol) was dissolved in dichloromethane dry (15 mL) and silver acetate (0.12 mmol) was added. The mixture was stirred for 3 h at 0 °C, excluding light. Then, the suspension was filtered through a pad of Celite to remove AgCl. The solvent was evaporated giving pure complex as powder.

*Bis [(N-methyl, N’-[(2-sodium alcoholate-2-phenyl-ethyl)imidazole-2-ylidine]gold(I)] acetate (***2***)*, Yield 45%. ^1^H NMR (400 MHz, CDCl_3_, *δ* ppm): 7.34 (m, 5H, Ph ring), 6.89 (d, 1H, NC*H*CHN), 6.86 (d, NCHC*H*N), 5.22 (m, 1H, C*H*O^−^), 4.46-4.40 (m, 2H, NC*H*_2_), 3.79 (s, 3H, NC*H*_3_), 1.98 (s, 3H, OCOC*H*_3_). ^13^C NMR (100 MHz, CDCl_3_, *δ* ppm): 176.1 (CH_3_*C*OO) 171.0 (N*C*N), 141.7 (*ipso* carbon aromatic ring), 128.6, 126.3, 126.1 (aromatic carbons), 123.2, 121.2 (backbone carbons), 72.9 (*C*HO^−^), 58.4 (N*C*H_2_), 38.1 (N*C*H_3_), 22.5 (*C*H_3_COO). [ESI-MS] = *m*/*z* 612.9 Dalton attributable to [C_22_H_17_AuN_4_O_2_Na_2_]^+^.

*Bis [(N-methyl, N’(2-methoxy-2-phenyl-ethyl)imidazole-2-ylidine]gold(I)] acetate* (**4**), Yield 55%. ^1^H NMR (400 MHz, DMSO-*d*_6_, *δ* ppm): 7.35 (m, 7H, Ph ring + NC*H*C*H*N), 4.76 (m, 1H, C*H*OCH_3_), 4.24 (m, 2H, NC*H*_2_), 3.69 (s, 3H, NC*H*_3_), 3.06 (s, 3H, OCH_3_), 1.78 (s, 3H, OCOC*H*_3_). ^13^C NMR (100 MHz, DMSO-*d*_6_, *δ* ppm): 174.6 (O*C*OCH_3_), 162.1 (N*C*N), 138.0 (*ipso* carbon of aromatic ring), 128.6, 128.3, 126.8 (aromatic carbons), 122.6, 122.3 (backbone carbons), 82.1 (*C*HOCH_3_), 56.4 (O*C*H_3_) 55.8 (N*C*H_2_) 37.7 (N*C*H_3_), 24.1 (*C*H_3_COO). [ESI-MS] = *m*/*z* 629.22 Dalton attributable to [C_26_H_32_AuN_4_O_2_]^+^.

*Bis [N-methyl, N’(2hydroxy-2-phenyl-ethyl)imidazole-2-ylidine]gold(I)] acetate* (***5***), Yield 50%. ^1^H NMR (400 MHz, DMSO-*d*_6_, δ ppm): 7.46-7.30 (m, 7H, Ph ring + NC*H*C*H*N), 5.10 (m, 1H, C*H*OH), 4.15 (m, 2H, NC*H*_2_), 3.74 (s, 3H, NC*H_3_*), 1.82 (s, 3H, OCOC*H*_3_). ^13^C NMR (100 MHz, DMSO-*d*_6_, *δ* ppm): 174.6 (O*C*OCH_3_), 161.8 (N*C*NO), 142.1 (*ipso* carbon of aromatic ring), 128.2, 127.5, 126.0 (aromatic carbons), 122.9, 122.0 (N*C*H*C*HN), 72.3 (*C*HOH), 57.6 (N*C*H_2_) 37.6 (N*C*H_3_), 24.1 (*C*H_3_COO). MALDI-MS = *m*/*z* 601.19 Dalton attributable to [C_24_H_28_AuN_4_O_2_]^+^.

*Bis [4,5-dichloro-(N-methyl, N’(2-hydroxy-2-phenyl-ethyl)imidazole-2-ylidine]gold(I)] acetate* (**6**), Yield 60%. ^1^H NMR (400 MHz, DMSO-*d*_6_, *δ* ppm): 7.42 (m, 5H, Ph ring), 5.84 (br, 1H, O*H*), 5.20 (m, 1H, C*H*OH), 4.22 (m, 2H, NC*H*_2_), 3.82 (s, 3H, NC*H_3_*), 1.84 (s, 3H, COC*H*_3_). ^13^C NMR (100 MHz, DMSO-*d*_6_, *δ* ppm): 174.8 (O*C*OCH_3_), 163.9 (N*C*N), 141.4 (*ipso* carbon of aromatic ring), 128.3, 127.7, 125.9 (aromatic carbons), 117.6, 116.5 (N*C*Cl*C*ClN), 72.2 (*C*HOH), 56.5 (N*C*H_2_) 37.7 (N*C*H_3_), 23.8 (*C*H_3_COO). MALDI-MS = *m*/*z* 739.07 Dalton attributable to [C_24_H_24_AuCl_4_N_4_O_2_]^+^.

### 4.2. LogP Value Determination

The logarithm of the partition coefficient (LogP) was evaluated by making a slight modification to the procedure reported in ref. [79]. Equal volumes of 1-octanol and water (distilled after milli-Q purification) were stirred for 20 h at room temperature. Then, they were separated to give 1-octanol-saturated water and water-saturated 1-octanol. Six (10, 20, 40, 50, 60 and 100 μM) standard concentrations of the compounds were prepared from the water-saturated 1-octanol. Analysis using UV/vis spectroscopy was used to obtain a calibration curve of absorbance vs. concentration. For each complex, a solution was prepared in the water-saturated 1-octanol (55 μM) and an equal volume of 1-octanol-saturated water was added. The solutions (biphasic) were mixed for 15 min and then centrifuged for 1h at 6500 rpm to allow separation.

Concentration in water-saturated 1-octanol was determined by UV-Vis spectroscopy. Reported LogP is defined as log[complex]_oct_/[complex]_H2O_.

### 4.3. Biology

#### 4.3.1. Cell Cultures

The five employed cell lines were purchased from American Type Culture Collection (ATCC, Manassas, VA, USA) and maintained as already reported [5]. MCF-7, MDA-MB-231, MCF-10A and Hek-293 were cultured, as indicated by Iacopetta et al. [5]. SkBr3 cells were maintained in DMEM high glucose, supplemented with 10% FBS and 100 μg/mL penicillin/streptomycin.

#### 4.3.2. MTT Assay

MTT assays [Sigma Aldrich (St.Louis, MO, USA)] were used in order to determine the in vitro anticancer activities of all the target compounds, as already reported [5].The compounds were tested at different concentrations (0.1-1-10-20-40 µM) for 72 h. Results are represented as percent (%) of basal and the IC_50_ values were calculated using GraphPad Prism 9 (GraphPad Software, La Jolla, CA, USA).

#### 4.3.3. Human Topoisomerase I (hTopo I) Relaxation Assay

Human topoisomerase I relaxation assays were carried out exposing the substrate, supercoiled pHOT1, to the recombinant human topo I (TopoGEN, Port Orange, FL, USA) and the most active compounds, following the manufacturer’s procedures (TopoGEN, Port Orange, FL, USA), with some adjustments [5].

#### 4.3.4. Human Topoisomerase II (hTopo II) Decatenation Assay

Human topoisomerase II decatenation assays were performed exposing the substrate, kinetoplast DNA (kDNA), to the human topoisomerase II (TopoGEN, Port Orange, FL) and the most active compounds, following the manufacturer’s procedures (TopoGEN, Port Orange, FL, USA), with some adjustments [5].

#### 4.3.5. Immunofluorescence Analysis

The MitoTracker Deep Red FM probe (MitoTracker Mitochondrion-Selective Probes, Invitrogen European Headquarters, Paisley, PA4 9RF, UK) was used for mitochondrial staining, as previously described [7]. For immunofluorescence analysis, the cells were seeded and further processed, as reported by Iacopetta et al. [7]. The primary antibodies used were: rabbit anti-β-Actin and vimentin (Santa Cruz Biotechnology, Dallas, TX, USA), mouse anti cytochrome c and NF-κB p65 (Abcam, Cambridge, UK), diluted 1:100 in bovine serum albumin (BSA) 2% overnight at 4 °C. The secondary antibodies were: Alexa Fluor^®^ 488 conjugate goat-anti-rabbit and Alexa Fluor^®^ 568 conjugate goat-anti-mouse (Thermo Fisher Scientific, MA, USA), diluted 1:500 and incubated for 2h at 37 °C. DAPI 0.2 μg/mL (Sigma Aldrich, Milan, Italy) was used for nuclei staining. A fluorescence microscope (Leica DM 6000) was used for fluorescence detection. All the images were acquired and processed using the LAS-X software.

#### 4.3.6. Actin Polymerization/Depolymerization Assay

The ability of the tested compounds to interfere with the actin polymerization and depolymerization reaction was measured using an Actin Polymerization/Depolymerization Assay Kit purchased from Abcam, following the manufacturer’s instructions. Both polymerization and depolymerization reactions occur in a 100 μL final volume, by using Labeled Rabbit Muscle Actin reconstituted with the Buffer G supplemented with 0.2 mM ATP and 0.5 mM DTT (1,4-dithiothreitol). For the polymerization assay, reconstituted actin was mixed with supplemented Buffer G and samples in a white 96-well plate and then the polymerization reaction was induced with the addition of the Buffer P supplemented with 10 mM ATP. The solution was mixed and the data acquisition started. For the Actin Depolymerization Assay, supplemented Buffers P and G were mixed and incubated in a white 96-well plate at room temperature for one hour to polymerize the actin, protected from light. Then samples were added and the data acquisition started. Latrunculin A and Cytochalasin B were used as control molecules at a concentration of 5 μM. For both the assays, the assembly of actin filaments was determined by measuring the fluorescence (Ex/Em: 365/410 nm) in kinetic mode for 1 h at room temperature using a microplate reader.

#### 4.3.7. Protein Lysate and Immunoblot Analysis

Protein lysates underwent the SDS-PAGE process, as already defined [80]. Then lysates were resolved on a 7.5% SDS polyacrylamide gel, transferred to a nitrocellulose membrane, and probed with antibodies against vimentin and GAPDH (Santa Cruz Biotechnology, Dallas, TX, USA). Then membranes were incubated with peroxidase-coupled goat anti-mouse IgG (Thermo Fisher Scientific, MA, USA) and the antigen-antibody complex was revealed using the ECL detection system (Amersham Pharmacia Biotech, Piscataway, NJ, USA). The images are representative of three independent experiments.

#### 4.3.8. Docking Studies

The crystal structures of the human Topoisomerase I in complex with DNA (PDB code 1A35) [81] and of Topoisomerase IIα in complex with a DNA fragment and etoposide (PDB Code 5gwk) [82] have been used as the bases to complete the three dimensional atomic models of human Topoisomerases (hTopo I and hTopo II), as previously described [7]. Furthermore, the crystal structure of the complex formed between the Beta/Gamma-Actin with Profilin and the acetyltransferase AnCoA-NAA80 [33] (PDB code 6nbw) was used as a target for the docking simulations. The formula of the compounds **1** and **6** have been designed, the coordinates of their atomic structures were determined and energy minimized using the program MarvinSketch (ChemAxon Ltd., Budapest, Hu) to evaluate the possible binding modes and the binding energies of our compounds, the above-mentioned proteins: the docking of the small molecules to the targets was done without a priori knowledge of the position of the binding site by the system. All the simulations were performed adopting the program’s default values. The protein and the ligands were prepared using the ADT graphical interface [83]. Polar hydrogens were added to each protein, and Kollman charged, assigned and solvation parameters were calculated. We considered our target proteins as rigid objects while all the ligands were handled as being completely flexible. We extended our search grid over the entire protein to properly calculate affinity maps. The search was carried out using a Lamarckian Genetic Algorithm where a population of 100 individuals was evolved for 100 generations using a mutation rate of 0.02. Analysis of the results was performed by ranking the different ligand poses accordingly to their binding energy. We gathered the results in clusters on the bases of the root mean squares deviation (RMSD) values of the position of each atom with respect to the corresponding of the starting geometry. We considered the molecule adopting the lowest energetic conformation in the most populated cluster as a most promising compound. In case two or more clusters were almost equipopulated and their energy distribution was spread, their corresponding molecules were considered as bad ligands [84]. 

Visual analysis of the lowest energy solutions for each cluster allowed us to identify the protein binding site. All the figures were drawn using the program Chimera [85].

#### 4.3.9. TUNEL Assay

TUNEL assay was employed to assess the cells’ apoptosis, following the manufacturer’s procedures (CF™488A TUNEL Assay Apoptosis Detection Kit, Biotium, Hayward, CA, USA). The cells were seeded and then further processed using the terminal deoxynucleotidyl transferase (TdT), as already described by Iacopetta et al. [7]. DAPI (Sigma, 0.2 µg/mL) was used for nuclei staining. A fluorescence microscope (Leica DM 6000) was used for fluorescence detection (20× magnification). All the images were processed using the LAS-X software. Images are representative of three independent experiments.

#### 4.3.10. Caspases Assay

The activities of caspases 3/7, 8, and 9 were measured by using the Caspase-Glo Assay according to the manufacturer’s guidelines (Caspase-Glo 3/7, 8, and 9 Assay Systems, Promega Corporation, Madison, WI, USA), as already described [7]. The luminescence was detected in a plate-reading luminometer (Synergy H1 Hybrid Reader, BioTek) for 30 min to 3 h (λ_ex/em_ = 490/570 nm). Results were reported as signal-to-noise ratio (background was determined from the culture medium without cells). The experiments were performed in triplicate.

#### 4.3.11. Statistical Analysis

Data were analyzed for statistical significance (*** *p* < 0.001, n.s. not significant, treated vs. CTRL) using one-way ANOVA followed by Dunnett’s test performed by GraphPad Prism 9. Standard deviations (SD) are shown.

## 5. Conclusions

Recently, *N*-heterocyclic carbenes (NHCs) have been shown to be versatile research tools, because of their chemical features making them suitable ligands for drug design in medicinal chemistry. In particular new NHC-gold complexes have been demonstrated to possess multiple mechanisms of action in cancer, interfering, for instance, with reductases, kinases, phosphatases, topoisomerases and cytoskeleton dynamics, inducing mitochondrial or DNA damage in cancer cells. Increasing literature data described many gold-NHC complex derivatives as effective anticancer agents, able to induce cell death by apoptosis with a low impact on the viability of normal cells. Amongst the new synthesized [NHC-Au(I)]Cl and [NHC-Au(I)]-OAc complexes, obtained with a green approach, we individuated the leads **1** and **6** as the most promising complexes, presenting a better anticancer and cytotoxic profile than *Cis*platin. By the means of in silico and in vitro studies, we proved that these complexes target directly at least three different intracellular proteins, namely the hTopo I and II, essential enzymes involved in DNA metabolism, and the actin, provoking a loss of the cytoskeleton function. The blockade of the above-mentioned protein functions induces the intrinsic apoptosis in both the types of breast cancer cells. Moreover, a regulation of vimentin levels and NF-kB intracellular localization has been observed, which is in agreement with the reported anticancer activity. These features make these complexes interesting anticancer compounds with a multitarget activity, useful for the development of potential drugs in breast cancer treatment.

## Figures and Tables

**Table 1:** Carbene carbons data for **1**–**8** complexes.

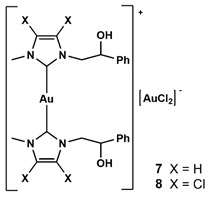	**Complex**	**Au-C (*δ*_C_) ppm**
	**1**	171.8 *^a^*
	**2**	171.0 *^a^*
	**3**	169.1 *^b^*
	**4**	162.1 *^b^*
	**5**	172.0 *^b^*
	**7** [13]	161.8 *^b^*
	**6**	170.2 *^b^*
	**8** [5]	163.9 *^b^*

Solvent *^a^* CDCl_3_ *^b^* DMSO-*d*_6_.

**Table 2:** Measured LogP values for NHC-Au(I) complexes.

Complex	LogP
**1**	0.079
**2**	0.047
**3**	0.62
**4**	0.68
**5**	0.12
**6**	0.78

**Table 3:** IC_50_ values of Au metal complexes (**1**–**6**), *Cis*platin and Latrunculin A, expressed in μM, related to their anticancer activity against different breast cancer cells (MDA-MB-231, MCF-7 and SkBr3) and cytotoxicity against normal cells (MCF-10A and Hek-293). The means ± standard deviations are shown.

IC_50_ (µM)					
Compounds	MDA-MB-231	MCF-7	SkBr3	MCF-10A	Hek-293
***Cis*platin**	28.7 ± 0.4	35.8 ± 0.7	7.4 ± 0.9	81.3 ± 0.6	16.3 ± 0.9
**Latrunculin A**	2.4 × 10^−2^ ± 0.9	0.14 ± 1.0	0.7 ± 1.2	4.5 × 10^−2^ ± 1.2	9.3 × 10^−2^ ± 0.6
**1**	15.8 ± 0.7	29.9 ± 1.1	83.0 ± 1.0	>200	>200
**2**	22.6 ± 1.1	30.6 ± 0.7	38.4 ± 0.4	39.6 ± 0.5	>200
**3**	2.2 ± 1.1	3.3 ± 1.4	10.9 ± 0.7	13.3 ± 1.0	39.0 ± 0.9
**4**	3.0 ± 0.7	9.4 ± 0.6	2.6 ± 0.7	5.2 ± 0.9	7.6 ± 1.0
**5**	58.5 ± 1.2	21.9 ± 0.5	58.9 ± 1.2	27.8 ± 0.7	>200
**6**	16.8 ± 1.2	1.2 ± 0.3	2.3 ± 0.9	24.4 ± 0.9	32.5±1.1

**Table 4:** Binding energies for both compounds **1** and **6** as resulting from in silico simulations, towards human topoisomerases I and II and actin.

Binding Energies (kcal/mol)			
**Compounds**	**h Topo I**	**h Topo II**	**Actin**
**1**	−8.42	−6.63	−8.09
**6**	−9.58	−8.65	−7.75

## Data Availability

Data is contained within the article and the Supplementary Materials.

## Supplementary Materials

The following are available online at https://www.mdpi.com/article/10.3390/ph15050507/s1, Figure S1 **A**: Bis [(*N*-methyl, *N*’-[(2-sodium alcoholate-2-phenyl-ethyl)imidazole-2-ylidine]gold(I)]acetate (2), **B**: Bis [(*N*-methyl, *N*’(2-metoxy-2-phenyl)ethyl)imidazole-2-ylidine]gold(I)] acetate (4), **C**: Bis [(*N*-methyl, *N*’(2-phenyl)ethyl)imidazole-2-ylidine]gold(I)] acetate (5), **D**: Bis [4,5-dichloro-(*N*-methyl, *N*’(2-hydroxy-2-phenyl)ethyl)imidazole-2-ylidine]gold(I)] acetate (6), **E**: Figure S1. Actin immunofluorescence studies in MCF-10A cells, **F**: Figure S2. Evaluation of vimentin expression levels in MCF-10A cells, **G**: Figure S3. TUNEL assay in MCF-10A cells, **H**: Figure S4. Caspases activity in MCF-10A cells, **I**: Figure S5. Mitochondria staining and cytochrome c detection in MCF-10A cells, **L**: Figure S6. NF-κB expression in MCF-10A cells.
