# Supplementary material for: Novel Au Carbene Complexes as Promising Multi-Target Agents in Breast Cancer Treatment
Source: Pharmaceuticals (Basel). 2022 Apr 21;15(5):507. doi: 10.3390/ph15050507 (PMC9146163; doi:10.3390/ph15050507)
Supplement: Supplementary file 1 [file pharmaceuticals-15-00507-s001.zip › pharmaceuticals-1661796-supplementary.pdf]

Novel Au carbene complexes as promising multitarget agents in breast cancer treatment

Jessica Ceramella, Annaluisa Mariconda, Marco Sirignano, Domenico Iacopetta, Camillo Rosano, Alessia Catalano, Carmela Saturnino, Maria Stefania Sinicropi and Pasquale Longo

List of contents

A: Bis [(N-methyl, N'-[(2-sodium alcoholate-2-phenyl-ethyl)imidazole-2-ylidine]gold(I)]acetate (2)........1

^{1}H-NMR of 2........1

^{13}C-NMR of 2........2

ESI-MS of 2........2

B: Bis [(N-methyl, N'(2-metoxy-2-phenyl)ethyl)imidazole-2-ylidine]gold(I)] acetate (4)........3

^{1}H-NMR of 4........3

^{13}C-NMR of 4........4

ESI-MS of 4........4

C: Bis [(N-methyl, N'(2-phenyl)ethyl)imidazole-2-ylidine]gold(I)] acetate (5)........5

^{1}H-NMR of 5........5

^{13}C-NMR of 5........6

ESI-MS of 5........6

D: Bis [4,5-dichloro-(N-methyl, N'(2-hydroxy-2-phenyl)ethyl)imidazole-2-ylidine]gold(I)] acetate (6)........7

^{1}H-NMR of 6........7

^{13}C-NMR of 6........8

MALDI-ToF of 6........9

E: Figure S1. Actin immunofluorescence studies in MCF-10A cells........10

F: Figure S2. Evaluation of vimentin expression levels in MCF-10A cells........11

G: Figure S3. TUNEL assay in MCF-10A cells........12

H: Figure S4. Caspases activity in MCF-10A cells........13

I: Figure S5. Mitochondria staining and cytochrome c detection in MCF-10A cells........14

L: Figure S6. NF-κB expression in MCF-10A cells........15

A: Bis [(N-methyl, N'-[(2-sodium alcoholate-2-phenyl-ethyl)imidazole-2-ylidine]gold(I)]acetate (2)
Yield 45%.

^{1}H-NMR of 2

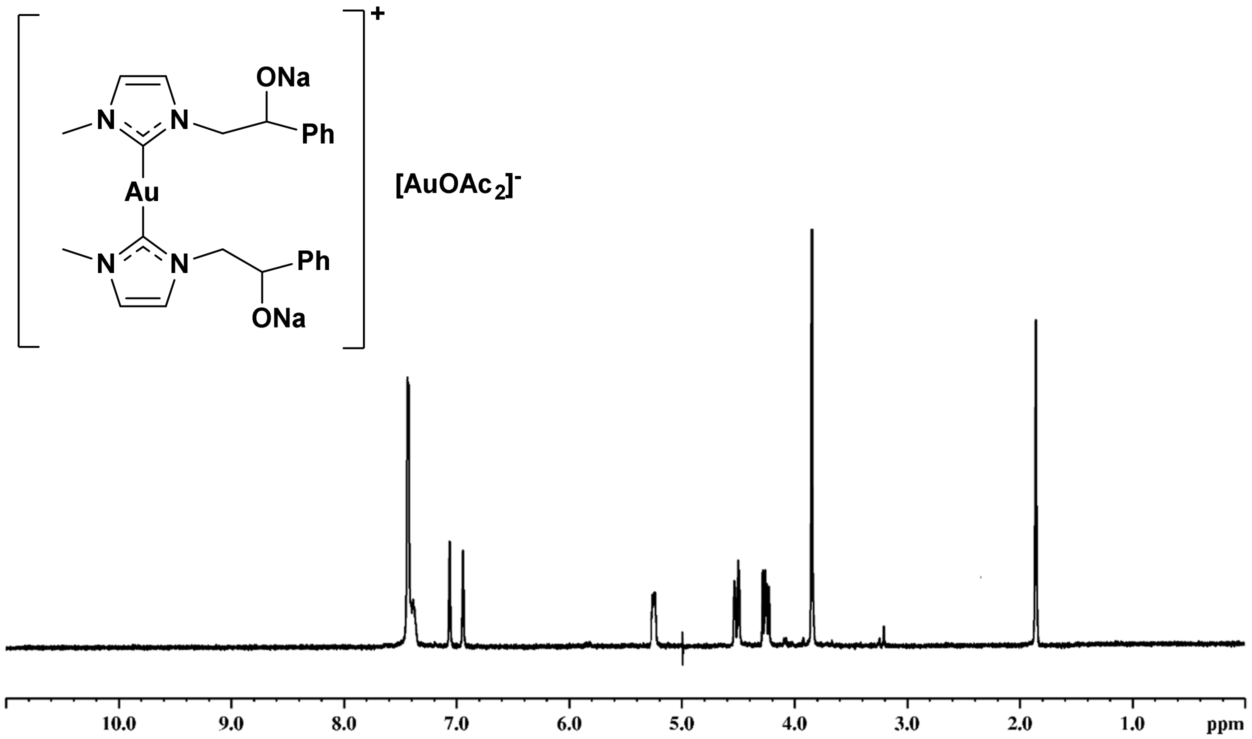

^{1}H NMR (400 MHz, $CDCl_3$, δ ppm): 7.34 (m, 5H, Ph ring), 6.89 (d, 1H, NC*H*CHN), 6.86 (d, NCHC*H*N), 5.22 (m, 1H, C*H*O$^-$), 4.46-4.40 (m, 2H, NC*H*$_2$), 3.79 (s, 3H, NC*H*$_3$), 1.98 (s, 3H, OCOC*H*$_3$).

^{13}C-NMR of 2

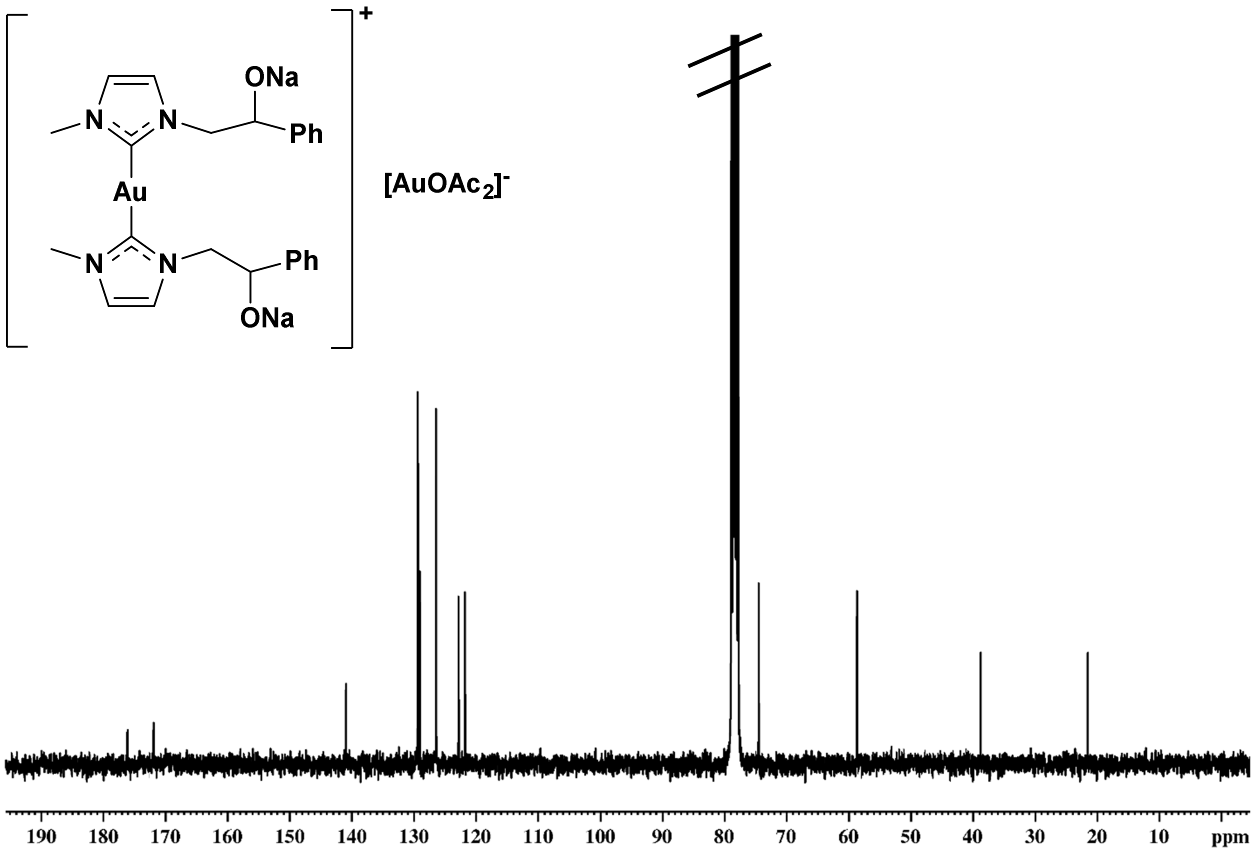

^{13}C-NMR (100 MHz, $CDCl_3$, δ ppm): 176.1 (CH_3*C*OO) 171.0 (N*C*N), 141.7 (ipso carbon aromatic ring), 128.6, 126.3, 126.1 (aromatic carbons), 123.2, 121.2 (backbone carbons), 72.9 (*C*HO^-), 58.4 (N*C*H_2), 38.1 (N*C*H_3), 22.5 (*C*H_3COO).

ESI-MS of 2

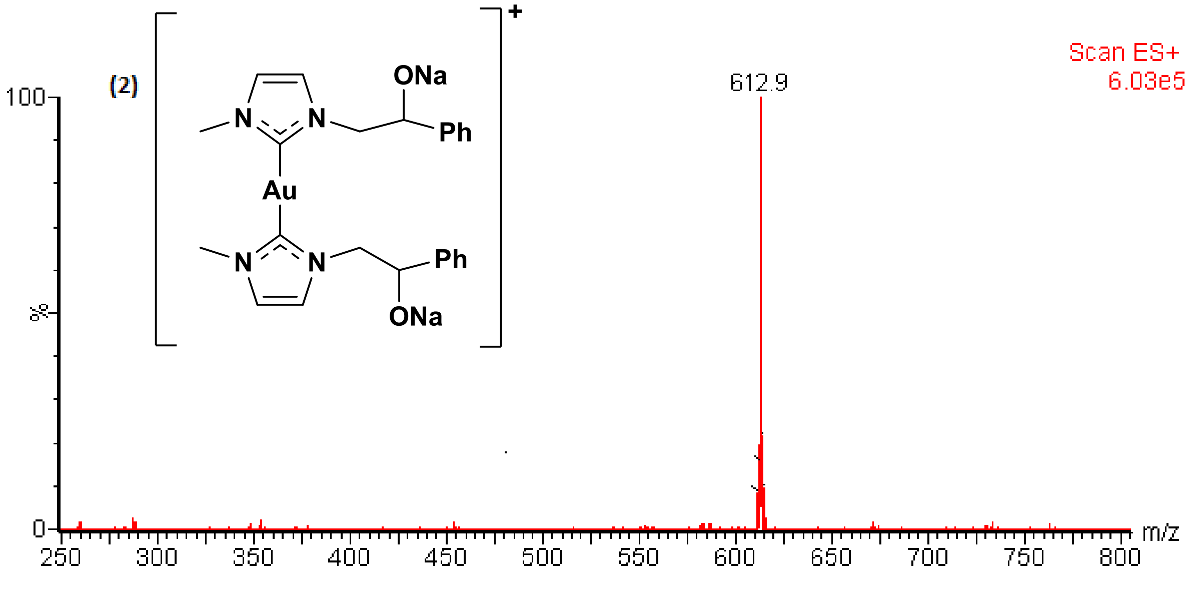

[ESI-MS] = m/z 612.9 Dalton attributable to $[C_{22}H_{17}AuN_4O_2Na_2]^+$.

B: Bis [(N-methyl, N’(2-metoxy-2-phenyl)ethyl)imidazole-2-ylidine]gold(I)] acetate (4)

Yield 55%.

^{1}H-NMR of 4

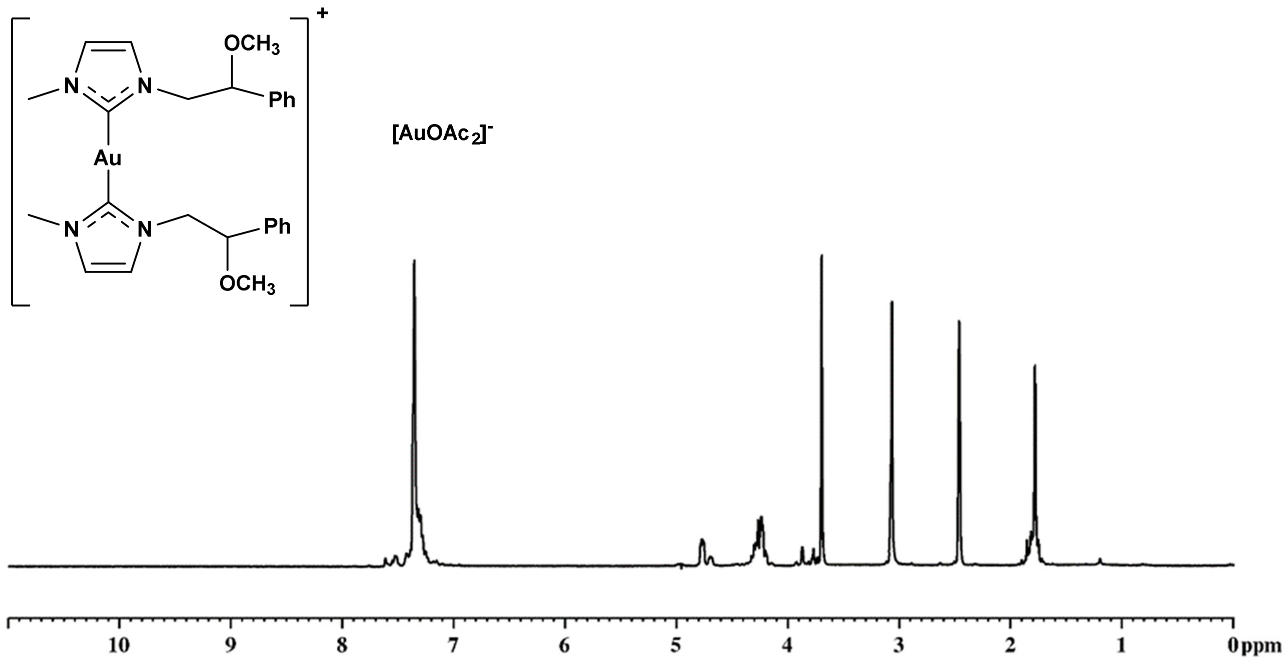

^{1}H-NMR (400 MHz, DMSO-d$_6$, δ ppm): 7.35 (m, 7H, Ph ring + NC*H*C*H*N), 4.76 (m, 1H, C*H*OCH$_3$), 4.24 (m, 2H, NC*H$_2$*), 3.69 (s, 3H, NC*H$_3$*), 3.06 (s, 3H, OC*H$_3$*), 1.78 (s, 3H, OCOC*H$_3$*).

^{13}C-NMR of 4

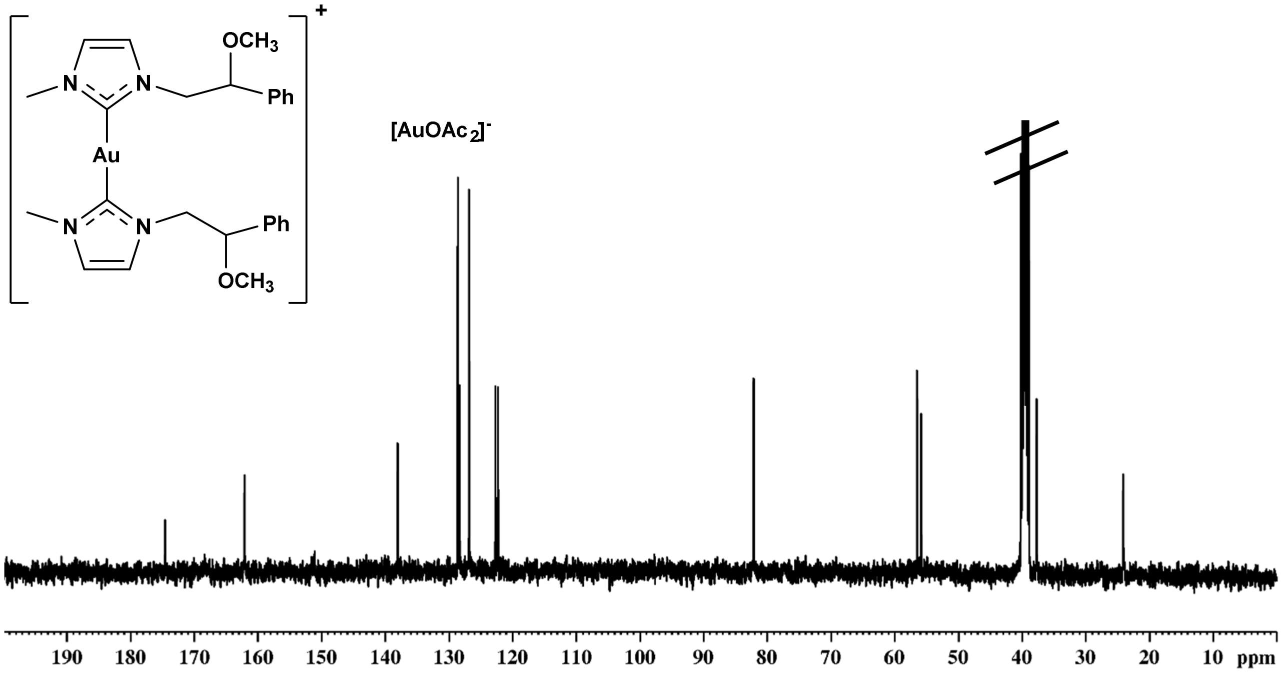

^{13}C-NMR (100 MHz, DMSO-d$_6$, δ ppm): 174.6 (O*C*OCH$_3$), 162.1 (N*C*N), 138.0 (ipso carbon aromatic ring), 128.6, 128.3, 126.8 (aromatic carbons), 122.6, 122.3 (backbone carbons), 82.1 (*C*HOCH$_3$), 56.4 (O*C*H$_3$), 55.8 (N*C*H$_2$), 37.7 (N*C*H$_3$), 24.1 (*C*H$_3$COO).

ESI-MS of 4

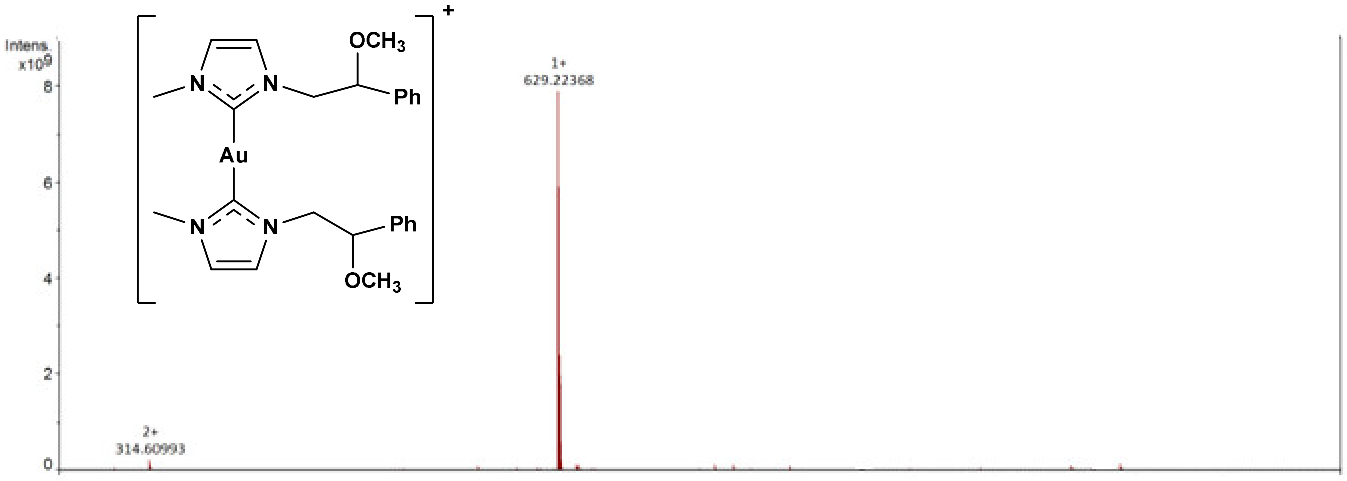

[ESI-MS] = m/z 629.22 Dalton attributable to [$C_{26}H_{32}AuN_4O_2$]$^+$.

C: Bis [(N-methyl, N'(2-phenyl)ethyl)imidazole-2-ylidine]gold(I)] acetate (5)
Yield 50%.

^{1}H-NMR of 5

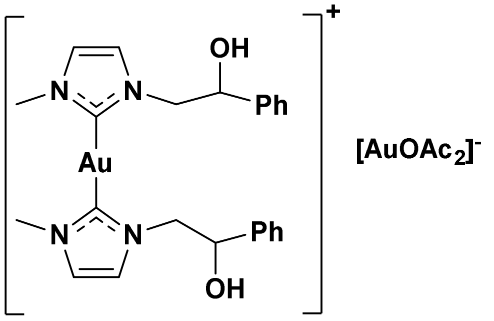

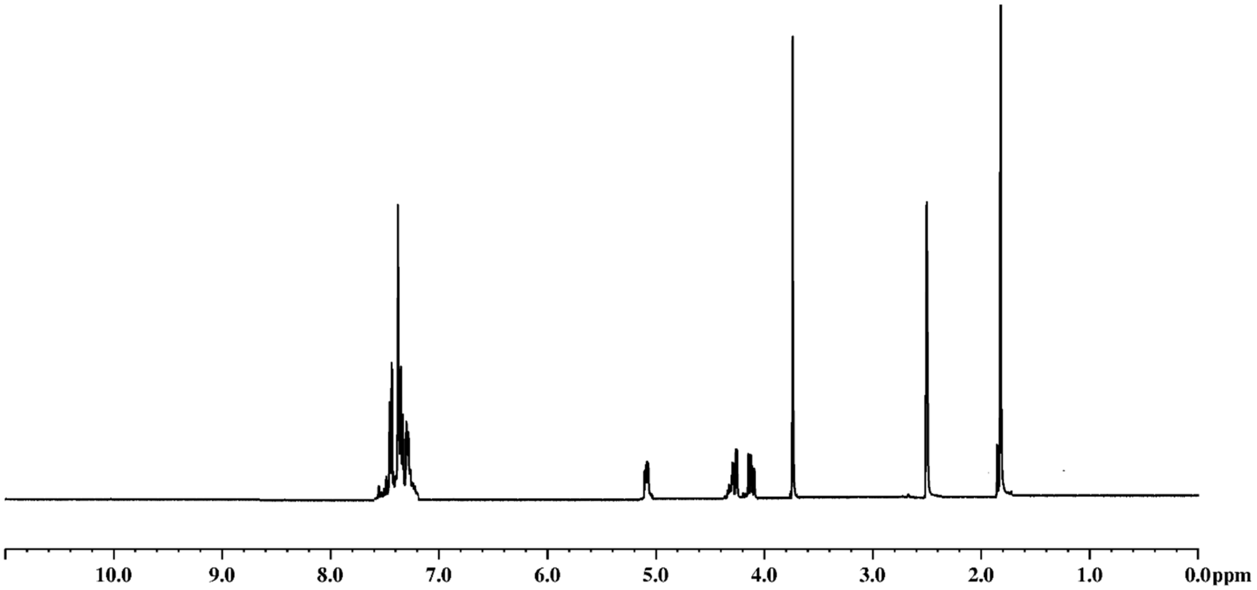

^{1}H-NMR (400 MHz, DMSO-d_6, δ ppm): 7.46-7.30 (m, 7H, Ph ring + NC*H*C*H*N), 5.10 (m, 1H, C*H*OH), 4.15 (m, 2H, NCH_2), 3.74 (s, 3H, NCH_3), 1.82 (s, 3H, OCOCH_3).

^{13}C-NMR of 5

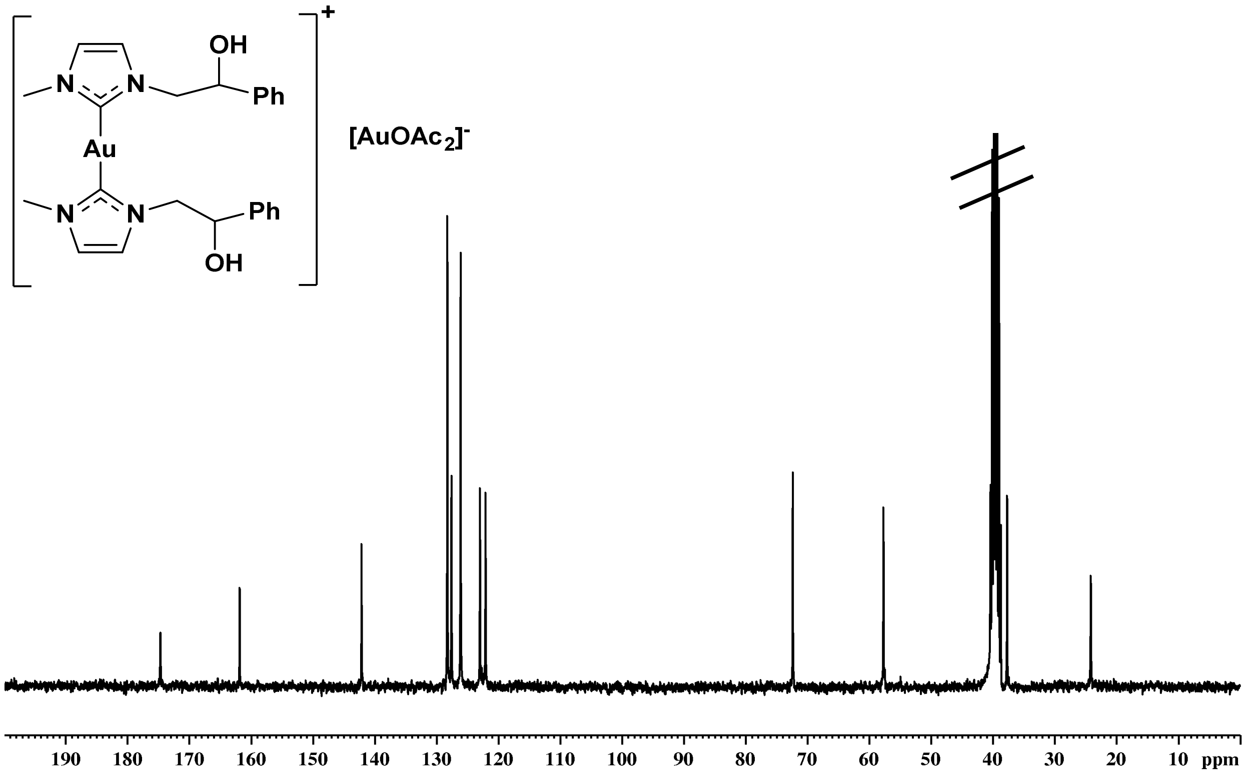

^{13}C-NMR (100 MHz, DMSO-d_6, δ ppm): 174.6 (O*C*OCH$_3$), 161.8 (N*C*N), 142.1 (ipso carbon aromatic ring), 128.2, 127.5, 126.0 (aromatic carbons), 122.9, 122.0 (backbone carbons), 72.3 (*C*HOH), 57.6 (N*C*H$_2$), 37.6 (N*C*H$_3$), 24.1 (*C*H$_3$COO).

ESI-MS of 5

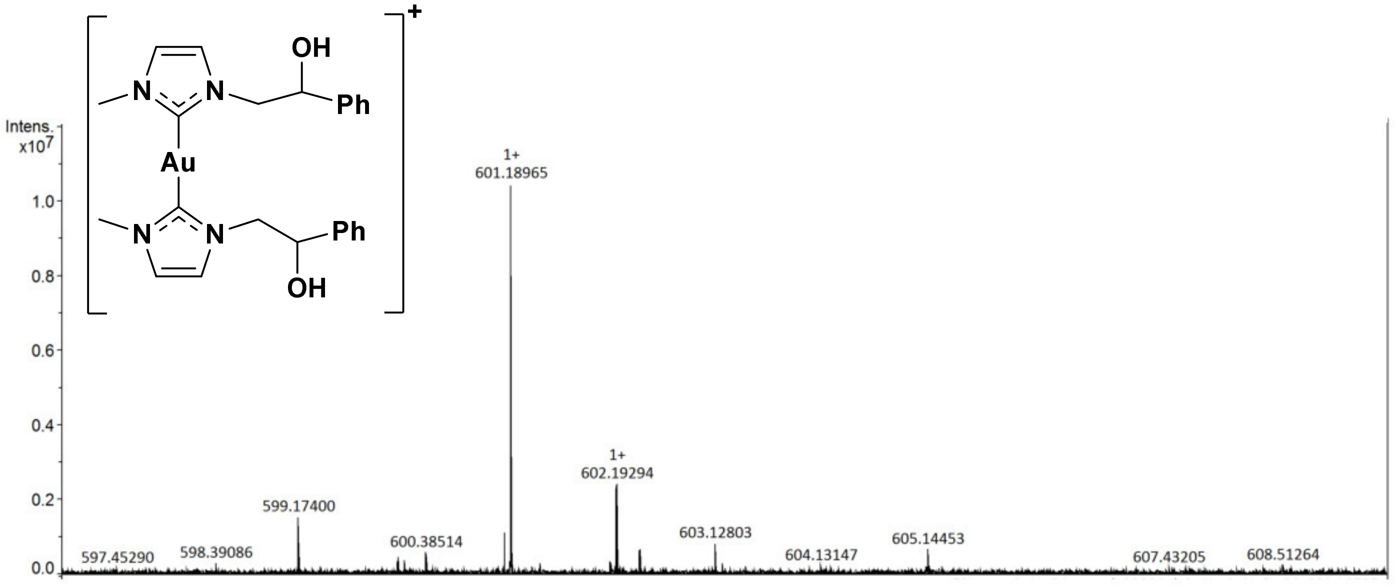

ESI-MS = m/z 601.19 Da attributable to $[C_{24}H_{28}AuN_4O_2]^+$.

D: Bis [4,5-dichloro-(N-methyl, N'(2-hydroxy-2-phenyl)ethyl)imidazole-2-ylidine]gold(I)] acetate (6)
Yield 60%.

^{1}H-NMR of 6

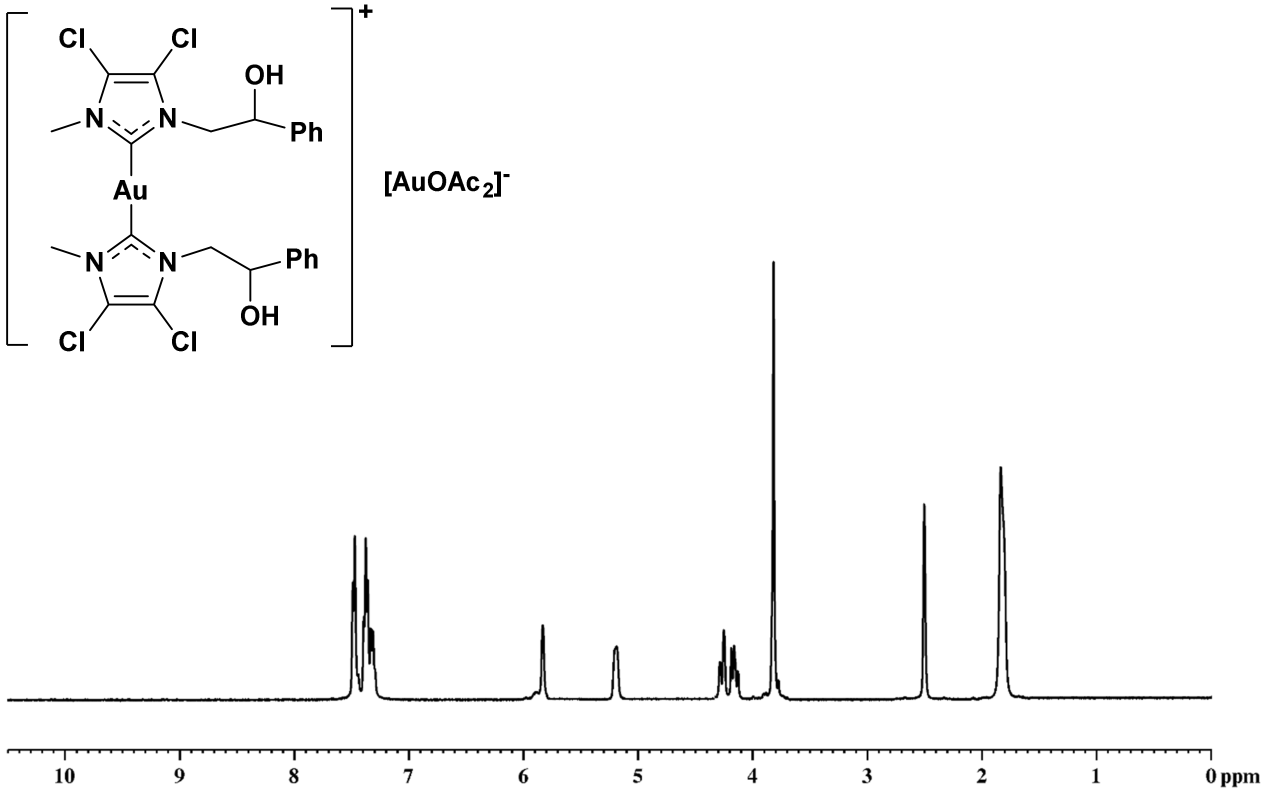

^{1}H-NMR (400 MHz, DMSO-d$_6$, δ ppm): 7.42 (m, 5H, Ph ring), 5.84 (s, 1H, O*H*), 5.20 (m, 1H, C*H*OH), 4.22 (m, 2H, NC*H*$_2$), 3.82 (s, 3H, NC*H*$_3$), 1.84 (s, 3H, COC*H*$_3$).

^{13}C-NMR of 6

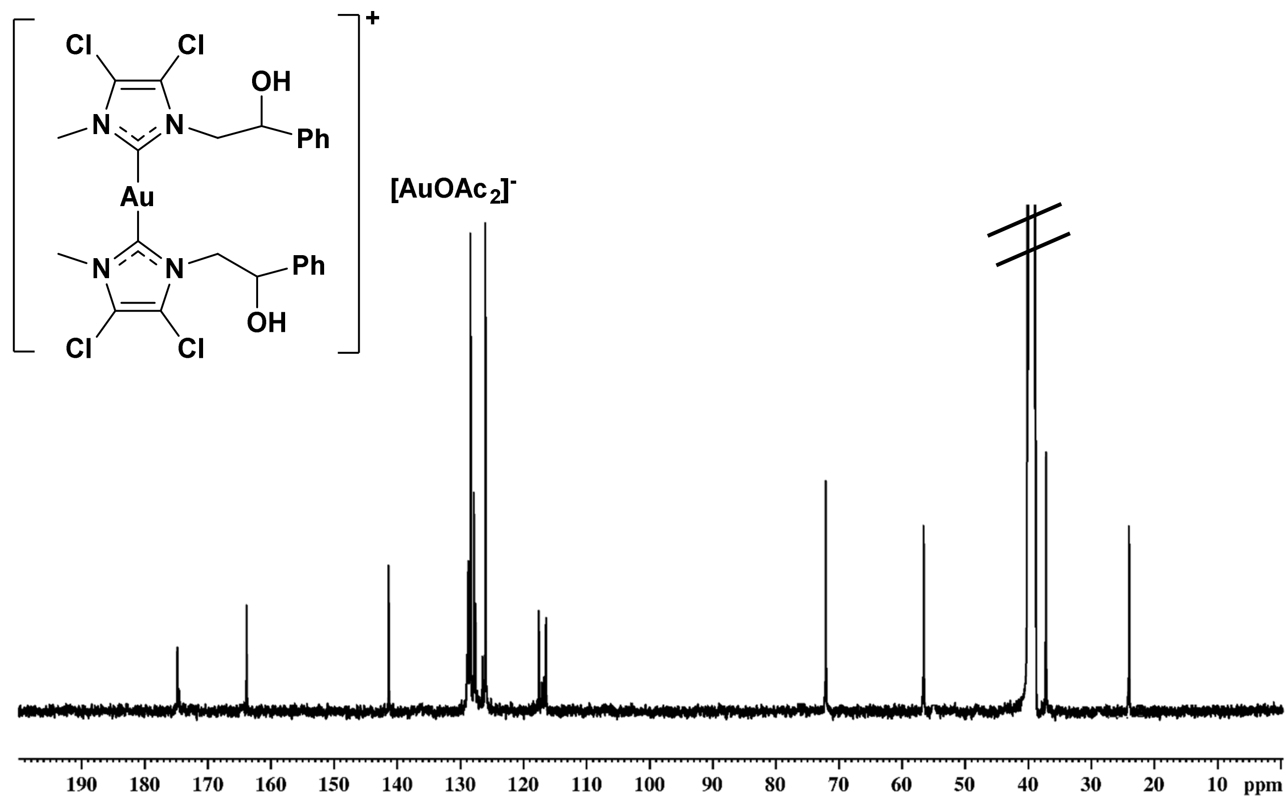

^{13}C-NMR (100 MHz, DMSO-d$_6$, δ ppm): 174.8 (O*C*OCH$_3$), 163.9 (N*C*N), 141.4 (ipso carbon aromatic ring), 128.3, 127.7, 125.9 (aromatic carbons), 117.6, 116.5 (backbone carbons), 72.2 (*C*HOH), 56.5 (N*C*H$_2$), 37.7 (N*C*H$_3$), 23.8 (*C*H$_3$COO).

MALDI-ToF of 6

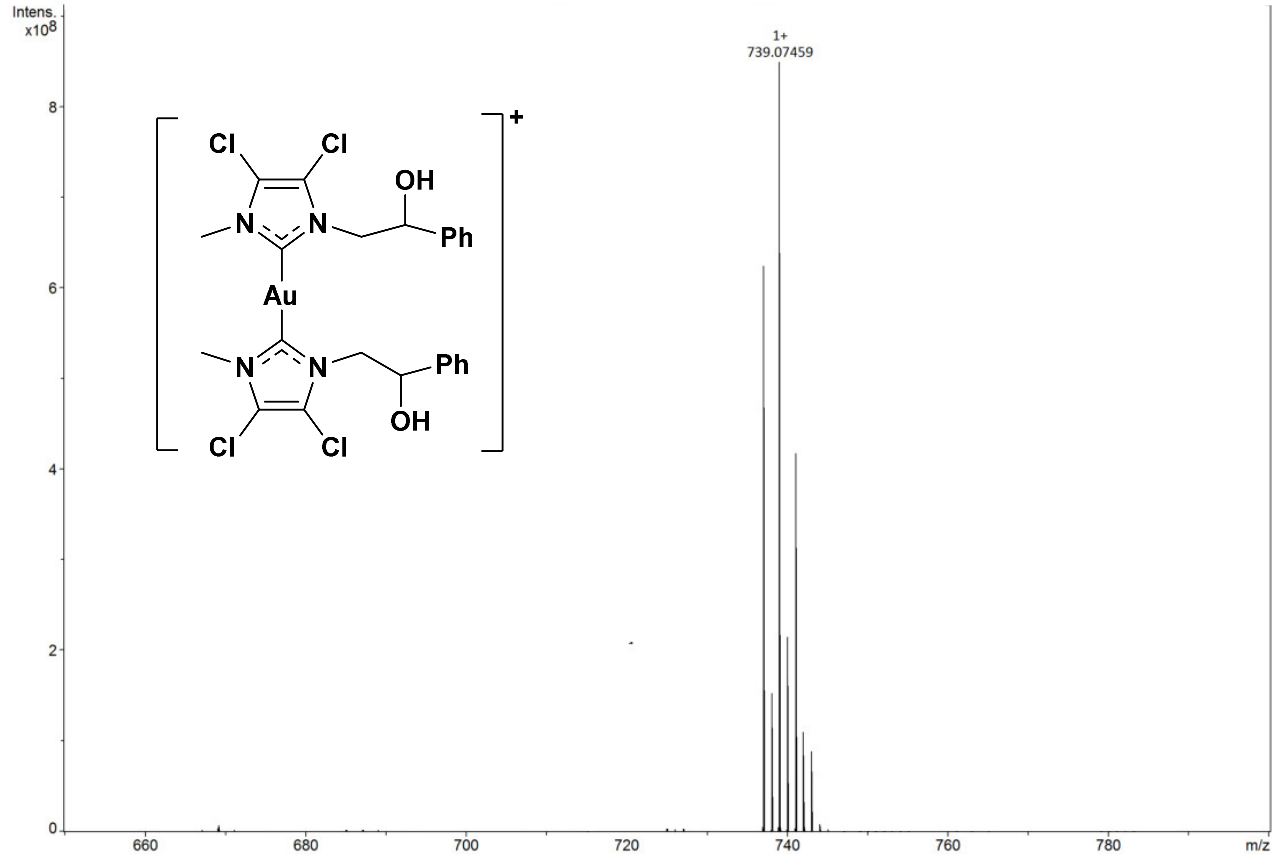

MALDI-MS = m/z 739.07 Da attributable to $[C_{24}H_{24}AuCl_4N_4O_2]^+$.

E: Figure S1. Actin immunofluorescence studies in MCF-10A cells

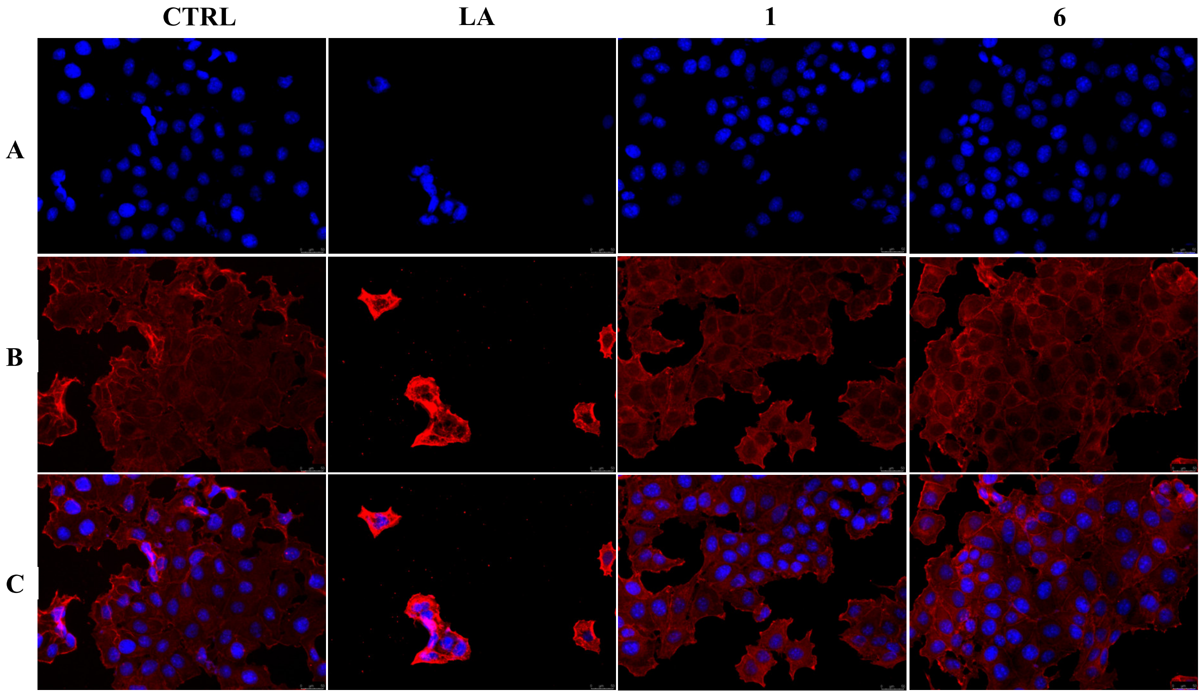

Figure S1. Actin immunofluorescence studies. MCF-10A cells were treated with compounds **1** and **6** (used at their IC_{50} value), with 0.1 μM Latrunculin A (LA) or with a vehicle (CTRL) for 24 h. After treatment, the cells were methanol fixed, incubated with primary and secondary antibodies, stained with DAPI and observed and imaged under the inverted fluorescence microscope at 20x magnification. CTRL and **1**,**6**-treated MCF-10A cells exhibited a normal arrangement and organization of the actin, instead, cells treated with LA showed an aberrant actin arrangement. Panels A: nuclear stain with DAPI ($\lambda_{ex}/\lambda_{em}$ = 350/460 nm); Panels B: β-actin (Alexa Fluor® 568; $\lambda_{ex}/\lambda_{em}$ = 644/665 nm); Panels C: show a merge. Representative fields are shown.

F: Figure S2. Evaluation of vimentin expression levels in MCF-10A cells

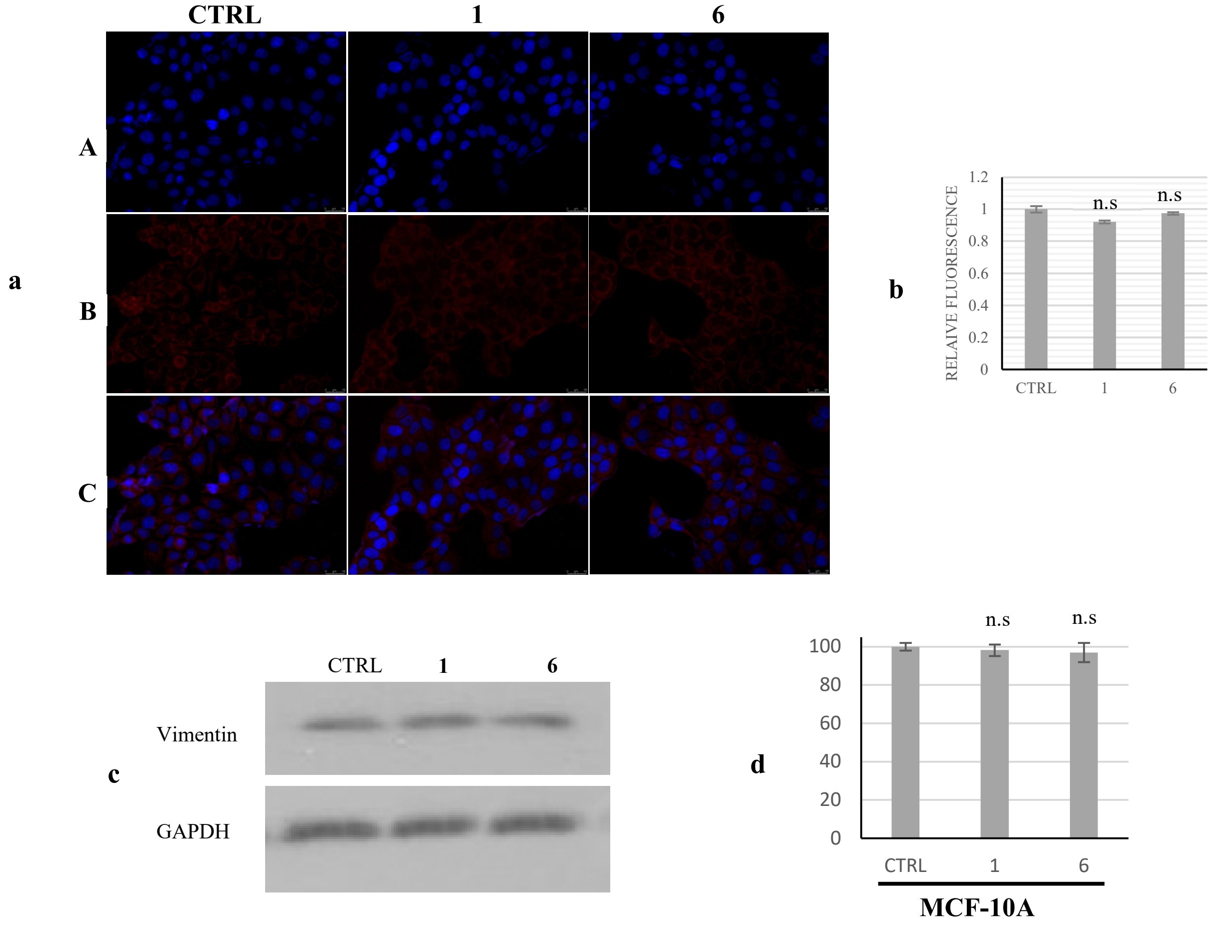

Figure S2. Evaluation of vimentin expression levels. (**a**) Immunofluorescence studies: MCF-10A cells were treated with compounds **1** and **6** respectively (used at their IC_{50} values) or with a vehicle (CTRL) for 24 h. After treatment, the cells were further processed, observed and imaged under the inverted fluorescence microscope at 20x magnification (for more details see Materials and Methods). No differences in the vimentin expression were recorded between the CTRL and compounds **1-6** treated cells. Panels A: nuclear stain with DAPI ($\lambda_{ex}/\lambda_{em}$ = 350/460 nm); Panels B: vimentin (Alexa Fluor® 568; $\lambda_{ex}/\lambda_{em}$ = 644/665 nm); Panels C: merge. Images are representative of three separate experiments. (**b**) Fluorescence quantification carried out using ImageJ; n.s. not significant, CTRL *vs* treated. (**c**) Western blots analysis: MCF-10A cells were treated with compounds **1** and **6** (used at their IC_{50} values) or with a vehicle (CTRL) for 24 h and then the total protein content was extracted and processed as reported in the Experimental Section. GAPDH: loading normalization. Images are representative of three separate experiments. (**d**) Western blots quantification carried out using ImageJ. n.s. not significant, treated *vs* CTRL.

G: Figure S3. TUNEL assay in MCF-10A cells

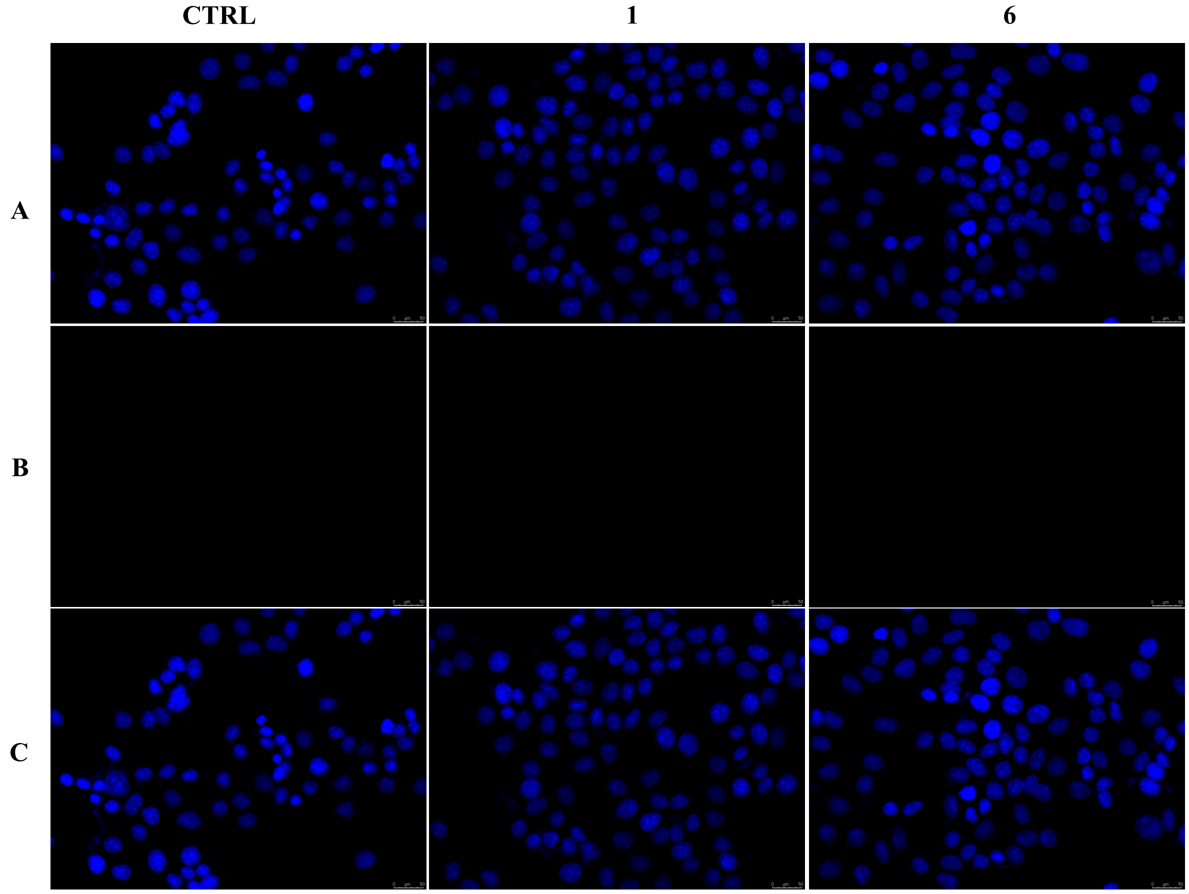

Figure S3. TUNEL assay. MCF-10A cells were treated with compounds **1** and **6** at the concentration equal to their IC_{50} or with vehicle (CTRL) for 24 h. Then they were exposed to the TdT enzyme and visualized under a fluorescence microscope (20x magnification). No apoptosis was detected. Panels A, DAPI $\lambda_{ex/em}$ 350 nm/460nm. Panels B, CF^{TM}488 A $\lambda_{ex/em}$ 490 nm/515nm. Panels C show the overlay channel.

H: Figure S4. Caspases activity in MCF-10A cells

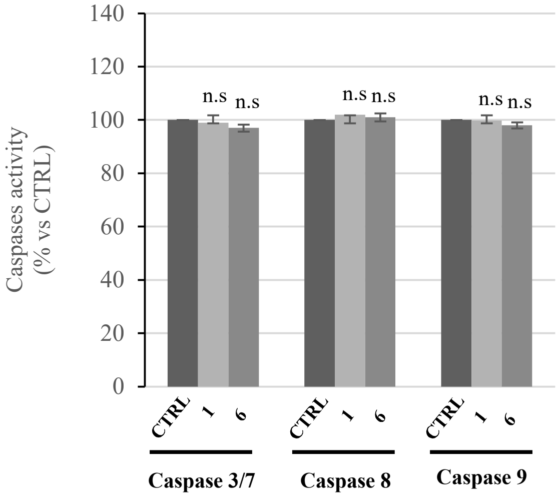

Figure S4. Caspases activity. No differences in caspases 3/7, 8 and 9 activity levels were detected between the vehicle-treated cells, used as control, and the cells treated with compounds **1** and **6**. Both the compounds were used at their IC_{50} values and incubated for 24 h. The data are representative of three independent experiments. n.s. not significant, CTRL *vs* treated.

I: Figure S5. Mitochondria staining and cytochrome c detection in MCF-10A cells

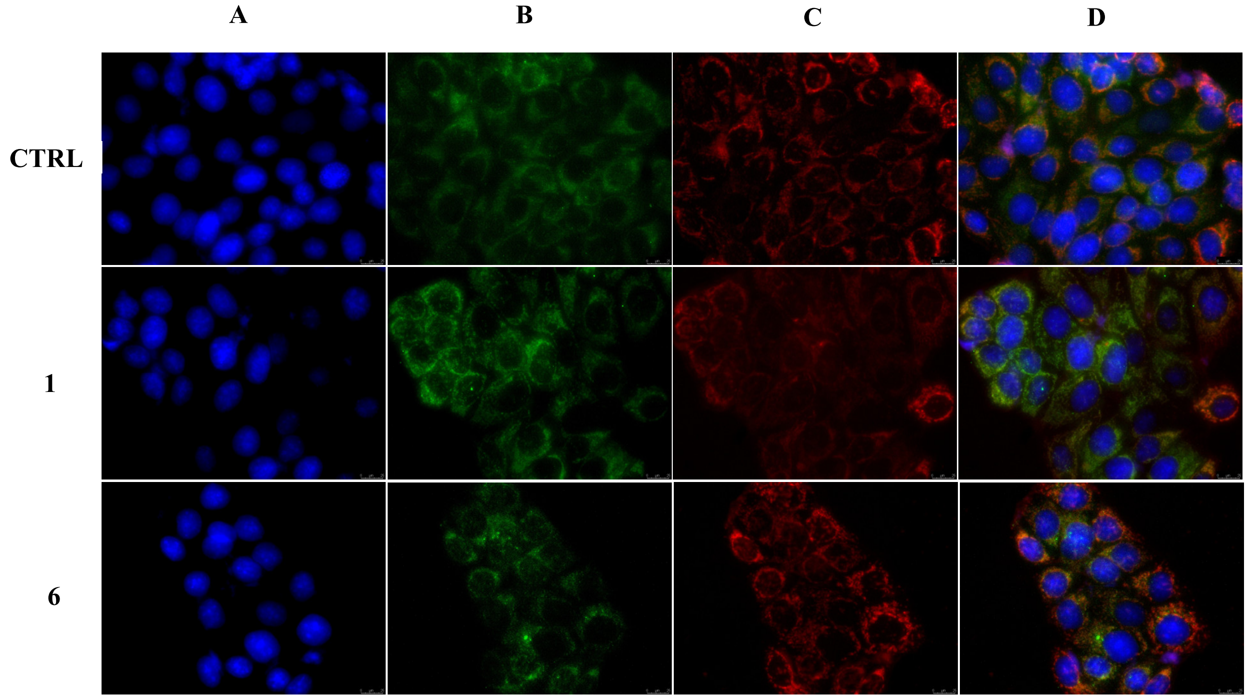

Figure S5. Mitochondria staining and cytochrome c detection in MCF-10A cells. **Figure S5.** Mitochondria staining and cytochrome c detection in MCF-10A cells. In the CTRL cells as well as in the cells treated with compound **1** and **6** at their IC_{50} value for 24 h, cytochrome c is localized within mitochondria. Panels A, DAPI $\lambda_{ex/em}$= 350/460 nm; Panels B, Alexa Fluor CF 488 $\lambda_{ex/em}$= 490/515 nm; Panels C, MitoTracker Deep Red FM probe $\lambda_{ex/em}$= 644/665 nm. Panels D: overlay channels. Images were acquired at 40x magnification and representative fields are shown.

L: Figure S6. NF-κB expression in MCF-10A cells

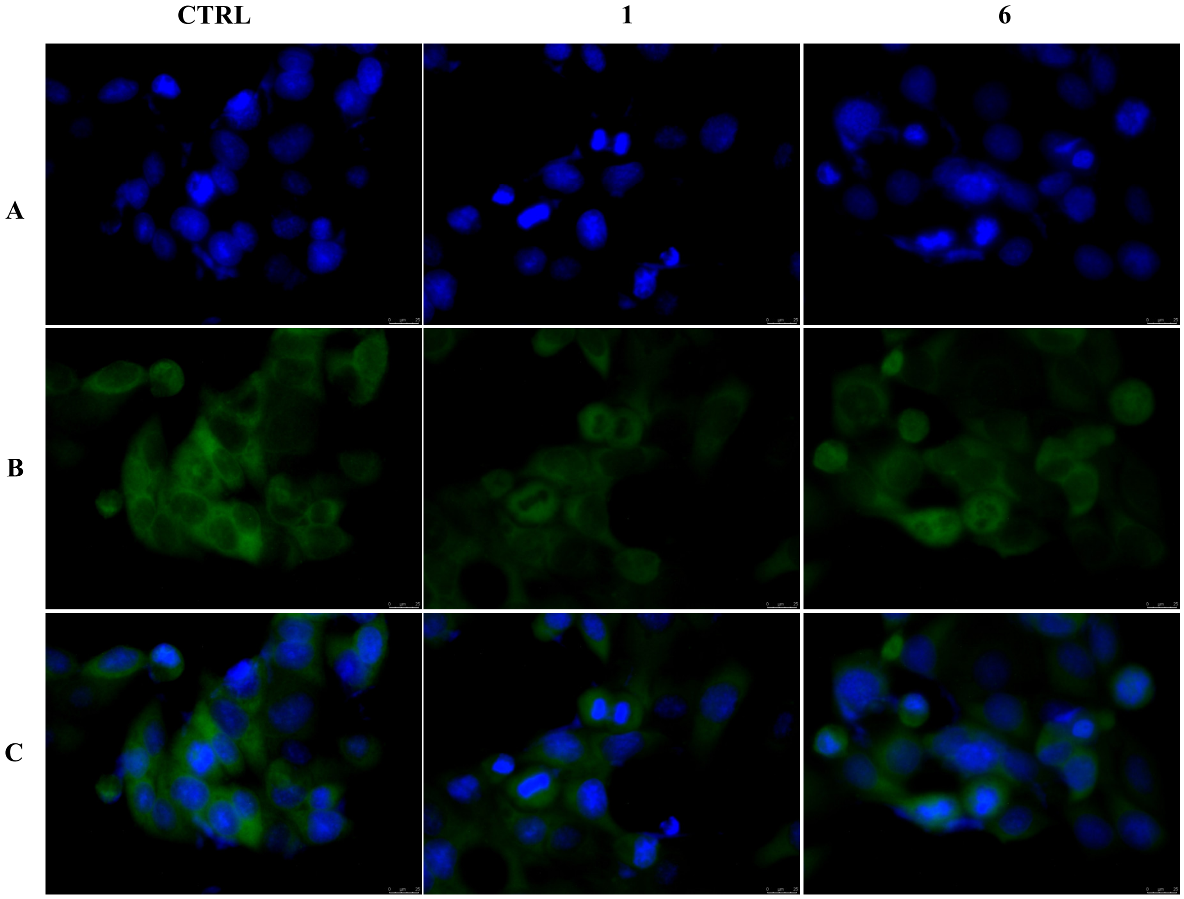

Figure S6. NF-κB expression in MCF-10A cells after treatment with compounds **1** and **6**. In the CTRL cells and in the cells exposed to compounds **1** and **6** NF-κB is localized within the cytoplasm and nucleus. Panels A, nuclear stain with DAPI ($\lambda_{ex/em}$= 350/460 nm); panels B, Alexa Fluor CF 488 ($\lambda_{ex/em}$= 490/515 nm); panels C, overlay channels. Images were acquired at 40x magnification and representative fields are shown.
